# Collaborative robots can augment human cognition in regret-sensitive tasks

**DOI:** 10.1093/pnasnexus/pgae016

**Published:** 2024-01-17

**Authors:** Millicent Schlafly, Ahalya Prabhakar, Katarina Popovic, Geneva Schlafly, Christopher Kim, Todd D Murphey

**Affiliations:** Mechanical Engineering, Northwestern University, Evanston, IL 60208, USA; Mechanical Engineering, Northwestern University, Evanston, IL 60208, USA; Mechanical Engineering, Northwestern University, Evanston, IL 60208, USA; Mechanical Engineering, Northwestern University, Evanston, IL 60208, USA; Mechanical Engineering, Northwestern University, Evanston, IL 60208, USA; Mechanical Engineering, Northwestern University, Evanston, IL 60208, USA

**Keywords:** collaborative robots, human–robot interaction, cognitive load theory

## Abstract

Despite theoretical benefits of collaborative robots, disappointing outcomes are well documented by clinical studies, spanning rehabilitation, prostheses, and surgery. Cognitive load theory provides a possible explanation for why humans in the real world are not realizing the benefits of collaborative robots: high cognitive loads may be impeding human performance. Measuring cognitive availability using an electrocardiogram, we ask 25 participants to complete a virtual-reality task alongside an invisible agent that determines optimal performance by iteratively updating the Bellman equation. Three robots assist by providing environmental information relevant to task performance. By enabling the robots to act more autonomously—managing more of their own behavior with fewer instructions from the human—here we show that robots can augment participants’ cognitive availability and decision-making. The way in which robots describe and achieve their objective can improve the human’s cognitive ability to reason about the task and contribute to human–robot collaboration outcomes. Augmenting human cognition provides a path to improve the efficacy of collaborative robots. By demonstrating how robots can improve human cognition, this work paves the way for improving the cognitive capabilities of first responders, manufacturing workers, surgeons, and other future users of collaborative autonomy systems.

**Significance Statement**

People will be using robots at home and in their jobs. During the creation of a robot, design choices are made regarding how a robot describes and accomplishes its goals. While robot behavior is known to impact human perception, here we show that robot design choices affect the human’s physiological cognitive availability and decision-making. Human cognition improves when the robots act more autonomously, managing more of their own behavior with fewer instructions from the human. Augmenting human cognition provides a path to improve the efficacy of collaborative robots. By demonstrating how robots can improve human cognition, this work paves the way for improving the cognitive capabilities of first responders, manufacturing workers, surgeons, and other future users of collaborative autonomy systems.

## Introduction

For decades, researchers have envisioned robots that enhance humanity by working collaboratively with humans. As robotic technology advances—with robots outperforming humans at both physical and computational tasks—a future with ubiquitous collaborative robots appears eminently achievable. However, despite exceeding the necessary technical requirements, robots have yet to demonstrate an unmitigated benefit to humans when there exists an alternative solution that does not rely on robots. For example, robot-assisted abdominopelvic surgery only reduces the frequency of surgical complications in 4 out of 50 randomized controlled trials, despite being less invasive (1). Robot-assisted upper-limb therapy with the MIT-Manus does not result in a statistically significant improvement in motor function compared to intensive therapy in a 12-week randomized controlled trial with 127 stroke patients (2), and results from lower-limb rehabilitation studies are similarly ambiguous (3–5). Lower-limb robotic prostheses do not result in a statistically significant improvement in energetic expenditure in a randomized crossover study with 12 amputees (6). Disaster relief robots generally perform tasks in areas too dangerous for humans instead of completing tasks alongside first responders (7–12). Robots on manufacturing floors or in homes rarely interact with humans. With no explanation for why collaborative robots are not producing the desired outcomes in the real world, it is unclear how the field should proceed.

Augmenting human cognition—that is, the human’s ability to reason—provides a path for improving collaboration outcomes. We demonstrate that robots can affect a human’s ability to reason, measuring participants’ physiological cognitive availability, and decision-making in a virtual-reality (VR) environment. The utility of current collaborative robots may be undermined by a reduction in the human’s capability to contribute to human–robot team performance. Several studies point to the relevance of cognition in powered wheelchair operation (13), prosthesis control (14, 15), and surgery (16). Although different areas of the brain govern different types of activities, such as motor control, memory, strategic planning, and sensory perception, prior work shows that high demands on one type of activity affects performance at another (17–19). We show that allowing the robot to act more autonomously, thereby reducing the communication requirements associated with using the robot, improves human cognition and allows the human to realize the benefits of robotic assistance.

By discovering that collaborative robots affect human cognition, this work introduces an unexplored research area at the intersection of robotics, computational neuroscience, and cognitive load theory that could impact the experience of all future users of autonomous systems. Although it is known that robots can influence human capability through facilitating the motor learning of prespecified tasks (20), augmenting human cognition allows the robot to improve human performance without knowing the task the human is attempting to accomplish. In many settings, we expect humans to possess knowledge about the task and how the task should be completed that is unavailable to the robot. We expect this work will prompt research that leverages our findings to develop collaborative robots that simultaneously improve cognitive availability and performance as well as research that uncovers new ways in which robot design affects human cognition. First responders, manufacturing workers, and surgeons, among others, may benefit from improved cognitive availability and decision-making.

### VR experimental platform

**Fig. 1.**
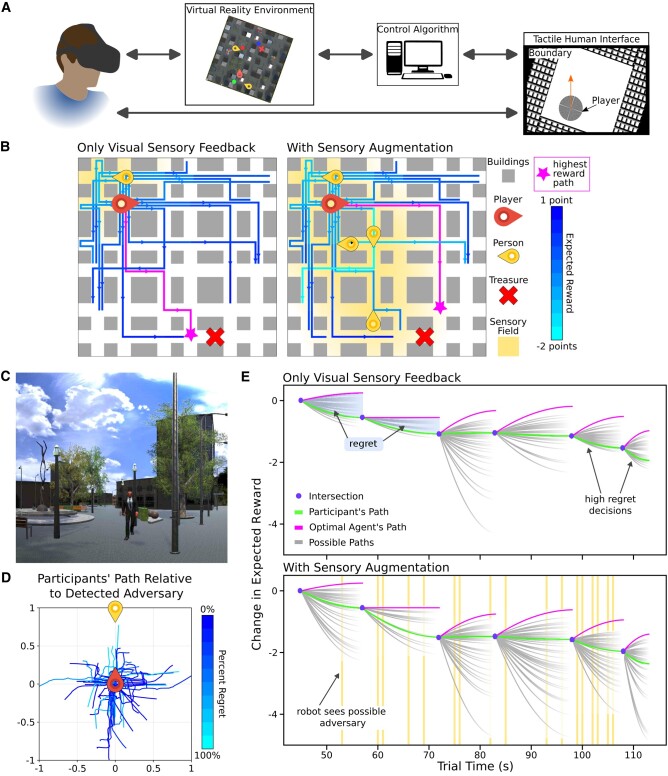
Sensory augmentation changes the regret landscape. The robots inform participants of possible adversaries in the environment, expanding the participant’s sensory field beyond the regions of the environment that are visually available. A) The experimental platform includes an HTC Vive headset, a VR environment, robot control algorithms, and a tactile human interface. B) Sensory augmentation alters the highest reward path through the environment. For each navigation decision, the optimal agent considers $4^{6}=4,096$ paths through the environment, determining how each path will affect the expected reward. C) The experimental task involves collecting treasures and avoiding adversaries. D) After detecting an adversary, participants generally move away from the adversary (right, left, or down); when participants move toward the adversary, they generally have high regret. Thirty-second paths are transformed to a coordinate system in which the adversary is detected at location (0,1) facing downward, and the start of the participant’s path is at location (0,0). E) We demonstrate the consequences of a sample participant’s decisions on the game reward according to the optimal agent. Yellow detections (where a robot sees a possible adversary) inform future decisions. With sensory augmentation, the optimal agent can anticipate more opportunities for a lost life, resulting in greater variation in how decisions are expected to affect game reward. Regret is the difference in expected reward between the decisions of the optimal agent and the decisions of the participant. We want the autonomy to help push the green line (the participant’s path) up as far as possible, minimizing regret.

At home and in their jobs, humans experience factors that influence their ability to make decisions—for example, time pressure (21), fear (22, 23), stress (24), and competing demands on their attention (25). When designing human studies, researchers typically minimize external factors to simplify study procedures and reduce experimental noise. However, in prior work, trends in cognitive load depend on the task demands experienced by the human (26–29). Here, we embrace the complexity inherent in real-world tasks and environments. Participants are immersed in a VR city environment (Fig. 1) and asked to collect treasure while being chased by adversaries patrolling the environment. Three robots augment participants’ sensory understanding of their environment by providing the locations of possible adversaries on a “minimap”—a map placed over part of the visual field of view. Participants provide instructions for the robots using a haptic tablet that can render spatially varying textures (30, 31), enabling the user to haptically localize themselves in the environment while their vision is occluded by the VR headset. VR can replicate features of real-world environments that may contain collaborative robots while providing a controlled experimental setting.

Control theory provides a framework for computationally interpreting the consequences of one’s actions in complex environments. We program an optimal agent to complete the VR task alongside the participant. At each intersection, the optimal agent chooses the navigation decision that will maximize expected game reward. Similar techniques have been used to beat international human champions in Chess and Go (32) as well as model animal (33, 34) and human behavior (35–40). The optimal agent’s decision is compared to the participant’s decision using a reinforcement learning concept called regret (41), visualized in Fig. 1. Participants experience regret if they could have made a better navigation decision based on available information. If a participant receives poor or incomplete information from the robots, it is still possible for the participant to make good, low-regret decisions. Since regret is measured relative to available information, regret controls for varying quantity or quality of information due to the human inputs or robot performance at information acquisition. Moreover, the optimal agent enables human performance at making decisions to be assessed separately from the entire human–robot team at receiving a high game score.

We conduct a human study where 25 participants with at least 1,000 h of video game experience complete 10, randomized, 5-min experimental trials. Each trial occurs in one of two VR environments that differ in building density. We compare four human–robot collaboration paradigms to a *no robots* condition in which the participant completes the task with no robot assistance. During *waypoint control*, the participant provides a path (i.e. a set of waypoints) for each robot to follow (8, 42). During *user coverage control*, the participant can command all three robots at once by providing a region of interest; the robots autonomously coordinate and collectively decide how to explore the user-defined region of interest (43). For *shared coverage control*, the robots consider both the high-priority regions of interest provided by the participant and high-priority regions identified autonomously by the robots (44). Lastly, during *fully autonomous coverage control*, the participant does not provide instructions for the robots, and the robots independently determine their coverage goals (45–49). The algorithms for specifying the coverage goals for each of the three aforementioned coverage control paradigms, including the shared specification for *shared coverage control*, are developed for this experiment, and the decentralized strategy for providing coverage of a distribution is adapted from Abraham and Murphey (45).

## Results

In this section, we evaluate whether the robot control paradigm affects human cognition. We hypothesize that the level of robot autonomy has a statistically significant effect on the number of instructions required to produce the desired behavior, the human’s cognitive availability, and the quality of human decision-making. We further hypothesize that robotic assistance will result in a statistically higher game score for paradigms associated with better human cognition.

### Physical interaction requirements decrease with increasing robot autonomy

**Fig. 2.**
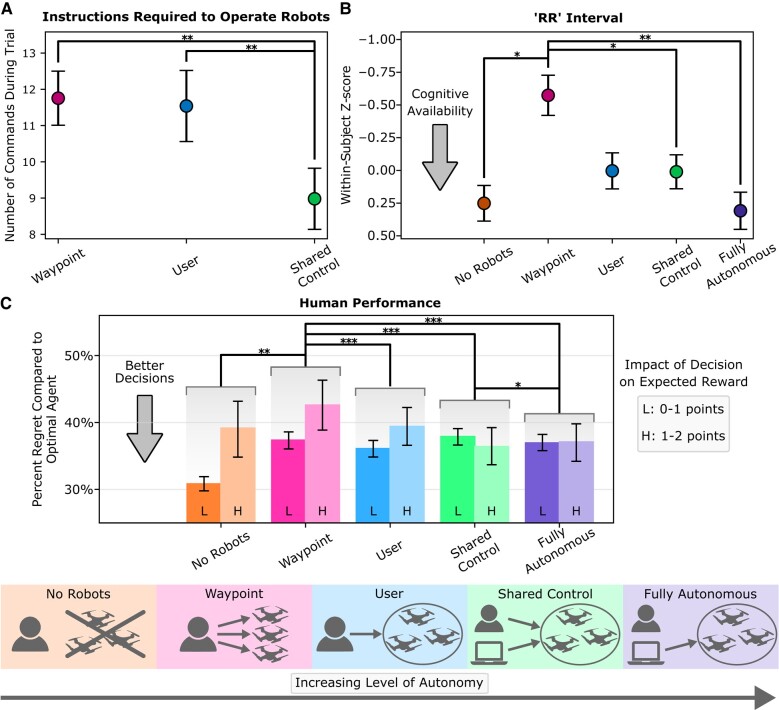
Effects of the level of autonomy on the human operator. As the level of autonomy increases, A) fewer instructions are required to produce the desired robot behavior, B) cognitive availability improves, and C) participants make better decisions, as found by comparing to the optimal agent’s decisions. Regret is the expected reward of the optimal agent’s decision minus the expected reward of the player’s decision (41) and is normalized here by the maximum impact of the decision on expected reward. We separate low-impact (L) and high-impact (H) decisions because the frequency of each type of decision differs for the different experimental conditions; the Materials and methods section describes how we account for this statistically. During high-impact decisions, one of the navigation options available to the participant will likely result in between 1-point and 2-point decrease in reward based on known information. Error bars indicate SE. Asterisks indicate statistical significance: ^*^P ≤ 0.05, ^**^P ≤ 0.01, ^***^P ≤ 0.001.

As the level of autonomy increases in Fig. 2A, fewer human instructions are necessary to produce the desired robot behavior. Level of autonomy has a statistically significant effect on the number of human commands provided during each trial (P<0.001, F(2,46)=10.21). *No Robots* trials with no robotic assistance and *fully autonomous coverage control* trials have zero physical interaction requirements.

During *waypoint control*, the human provides distinct paths (i.e. a set of sequential waypoints) for each robot to follow. Each waypoint input requires the human to execute a four-action procedure: (i) a double tap to indicate the start of an input, (ii) an action where the user indicates a path for the robot to follow, (iii) another double tap to indicate the end of the input, and (iv) an additional tapping action to indicate the robot for which the waypoints are intended. To update the behavior of all n=3 robots, *waypoint control* requires a total of 4×n=12 user actions. Due to the large physical interaction requirements, participants often chose to update only the behavior of one of the robots in response to changing game conditions.

When the robots are allowed to manage how the robot paths are specified during *user coverage control*, the physical interaction required to direct the behavior of all robots diminishes. One command consisting of three actions (one double tap to indicate the start of an input, an action where the user shades the regions of interest, and another double tap to send the command to the robots) updates the behavior of all robots. Unlike *waypoint control* where the physical interaction requirements scale linearly with the number of robots, one command indicating the regions of interest applies to an arbitrary number of robots.

During *shared coverage control*, the robots are allowed to contribute to a shared specification of the regions of interest. Using a computational model of the human’s sensory field and exact locations of previously detected people, the robots can anticipate some regions that are of interest to the human. Consequently, participants find that fewer commands are necessary to produce the desired robot behavior.

### Cognitive availability increases with increasing robot autonomy

To provide instructions for the robots, the user must devote cognitive resources to producing the contents of the instructions and operating the interface. When cognitive workload increases, the heart pumps faster to supply oxygen and other essential nutrients to the brain (50). Using an electrocardiogram (ECG), we compute participants’ average “RR” interval, the time between consecutive “R” peaks on an ECG signal, which is a more precise method of measuring heart rate or pulse. Although there are many other physiological measures of cognitive availability ranging from pupil diameter to skin conductivity (51), heart rate and its variants, such as the “RR” interval, are the most frequently reported physiological measure of cognitive availability (52). Due to the psychological biases associated with subjective measurements of cognitive availability (16), we do not use surveys like the NASA-TLX. Moreover, unlike other measures like the variation in the “RR” interval (often referred to as heart rate variability), the effect of cognitive workload on the “RR” interval is consistent for at least 1 h (53). A greater “RR” interval corresponds to more steady-state cognitive availability and more cognitive resources to make decisions and respond to unexpected events.

As the level of autonomy increases (Fig. 2B), participants have significantly more cognitive availability (P<0.001, F(4,88)=5.47). This result is consistent with prior work that uses a secondary task to measure cognitive availability (54). This trend can be largely explained by the physical interaction requirements of each human–robot paradigm. Participants have the least cognitive availability using *waypoint control*, the human–robot paradigm with the greatest physical interaction requirements. It is plausible that different types of commands (e.g. waypoints vs. shading) have different cognitive requirements; providing waypoints requires participants to reason from the robot’s perspective, which hurts performance at an assembly task (55, 56). By changing the structure of the human–robot interaction and allowing the robots to manage some or all of their own behavior using *shared coverage control* or *fully autonomous coverage control*, we significantly improve participants’ cognitive availability.

### Human decision-making improves with increasing robot autonomy

Improved cognitive availability implies that the human has more cognitive resources to dedicate to tasks besides operating the robots. We instruct participants to navigate through the environment such that they avoid adversaries and collect treasures, maximizing a reward quantity known to participants prior to the experiment as the final game score. To determine if the observed differences in cognitive availability influence human performance, we look at the discrete decisions made by participants at intersections in the environment, comparing the human’s decisions to the optimal agent’s decisions using the regret metric. The optimal agent learns from 10,000 forward simulations of the environment, where the optimal agent executes six randomly selected navigation actions in each simulation.

In Fig. 2C, we show that improved cognitive availability translates to better navigation decisions. Level of autonomy significantly affects decision regret (P<0.001, $\chi^{2}=30.20$). While many report participants’ perceived cognitive availability when using a robot (54, 57–59), we measure participants’ physiological cognitive availability and link changes in cognitive availability to the human’s performance separate from the performance of the entire human–robot team. Our finding that human decision-making can be impacted by robots means this research is urgently relevant to applications like disaster response and surgery, where human decision-making is critical to human lives.

### Only shared control improves game score compared to no robots

Since the robots provide participants with information relevant to task performance, it would be understandable to expect that performance would improve with robotic assistance. However, most robotic-assistance paradigms do not significantly change the game score compared to the *no robots* condition: *waypoint control* (P=0.842, t(47)=0.20), *user coverage control* (P=0.783, t(47)=  −0.28), or *fully autonomous coverage control* (P=0.972, t(47)=0.035). This result complements the wide range of recent studies that found no improvement in clinical outcomes due to robotic assistance (1–6).

The only control paradigm that resulted in a significantly different game score compared to *no robots* is *shared coverage control* (P=0.031, t(47)=−2.22), consistent with prior work showing the performance benefits of shared control paradigms (20, 59–63). Likewise, we find that level of autonomy has a statistically significant affect on game score due to superior performance using shared coverage control, as explained in Table S4. Human–robot team performance is a combination of human performance and robot performance. No paradigm resulted in statistically more cognitive availability than *shared coverage control*. While participants using *fully autonomous coverage control* have high cognitive availability and decision-making capabilities, fully autonomous robot behavior does not allow the human to communicate real-time coverage needs. The utility of robots in high-pressure scenarios depends on the format of the human–robot communication. By minimizing the cognitive load induced by using a robot, we demonstrate that robots can be helpful in achieving task goals.

## Discussion

Here, we show that robots can augment human cognition at an unknown task. This finding has implications for the physiological experience and capabilities of all future users of robots. By offering an explanation for why collaborative robots are not producing desired outcomes, this work provides a path for enabling humans to benefit from advances in robotic technology. In particular, impaired individuals reliant on a robot for mobility could use the additional cognitive availability to pursue hobbies, improving quality of life, or increase their own performance at the task, improving clinical outcomes.

Until now, the connection between robots and cognitive availability may have been obfuscated by a focus on relatively simple experimental tasks with few if any distractions. When a human is experiencing low-task demands, adding workload can improve cognition (26). For example, in the field of education, increasing students’ cognitive engagement and deceasing cognitive availability through interactive and active learning strategies improves performance at information retention (64). Similarly, for controlling the x−y position of a formation of robots, participants with less cognitive availability perform better (65). However, when the human is experiencing high-task demands and approaching their cognitive capacity, additional workload hurts cognition (26, 29). Moreover, as task demands increase, alleviating workload has a stronger effect on cognition (26–28). In line with prior work, we find that statistical trends strengthen for the more complex, low-density virtual environment compared to the high-density virtual environment as detailed in Fig. S11. In the low-density environment, there are more opportunities for high-regret decisions. To identify trends in cognition, it may be necessary to immerse participants in environments similar to the settings in which people will use robots.

By demonstrating that robot design, including the algorithms that determine how the robots describe and achieve their objective, affects the physiological state of the human operator—our work connects decades of research on cognitive load theory to the field of robotics. A robot’s hardware and software determine the format in which the robot can communicate with the human. Notable human–robot communication paradigms include tablets, natural language, gestures, programming interfaces, and human motion (66–68). Yet, improvements to the mechanical abilities of robots typically occur without the involvement of human users. To inform future robotics research, this work begins the process of developing guidelines for how one should expect robot design choices to affect a human’s physiological cognitive availability and performance.

While the goals of the treasure-gathering task presented here differ from many current and future tasks for collaborative robots—especially the clinical tasks motivating this work—our experiment is similar to a wide range of real-world settings in two important ways. Firstly, our task stresses participants’ cognitive capacity, which is a known consideration in powered wheelchair operation (13), prosthesis control (14, 15), and surgery (16). Secondly, the human participant is performing other tasks while operating the robot, concurrently navigating their own position in the environment and making strategic decisions about how to best use the robots. Additionally, our task is experimentally practical; it enables a straightforward and computable interpretation of the quality of each decision, allowing us to connect cognitive availability to improved human performance. The clinical tasks that form part of the motivation for this work do not have similar structure supporting assessment of decision-making (e.g. robot-assisted therapy does not necessarily take place on a grid). By demonstrating that it is possible for robots to augment human cognition and that augmenting human cognition can be an intermediate step toward improving human–robot collaboration outcomes, this work is paving the way for new strategies for augmenting cognition that are specific to rehabilitation, prostheses, surgery, manufacturing, search and rescue, disaster relief, or personal robots.

Our specific approach to improving human cognition could be applicable to surgeons and first responders. In both settings, the human is responsible for specifying the path of the robot, communicating both the task goal and how the robot should accomplish the task. During some robot-assisted surgeries, the surgeon controls the robot’s position as it physically probes different locations on an organ (69, 70). Similarly, following disasters such as the 2021 landslide in Norway (10) and Hurricane Harvey (7), among others (8, 11, 42), the first responders individually piloted the path of each robot, akin to the waypoint control paradigm in our study. By enabling robots to act more autonomously using the strategy proposed here, researchers may be able to improve the cognitive availability and decision-making of surgeons and first responders, whose cognitive performance is critical to human lives.

## Materials and methods

### VR environment

**Fig. 3.**
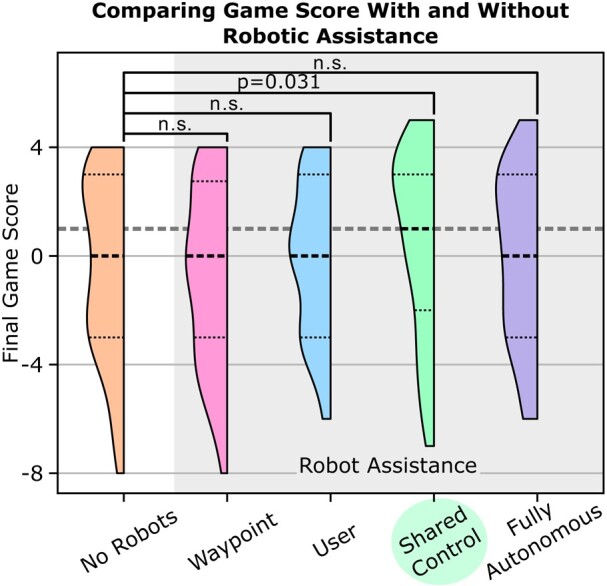
Despite the common assumption that robots should improve performance, the effect of robotic assistance on game score depends on the format of the human–robot interaction. Shared coverage control is the only assistance paradigm that simultaneously reduces human operations, improves cognitive availability, and improves decision-making, while incorporating the participant’s intent, explaining its advantage. The area of each shape is equal, and the relative width corresponds to the number of trials that resulted in a particular game score. The dotted lines indicate the quartiles of the distribution. “n.s.” indicates no statistical significance.

Two distinct VR environments are designed to portray areas of low-spatial visibility (high-building density) and high-spatial visibility (low-building density). For the low-density environment, 25% of the buildings are removed and replaced with outdoor features that might be found in a city, such as parks, outdoor dining, and public seating areas. Participants use an HTC Vive headset and controllers to maneuver in the VR environment created using Unity 3D software. So that the participant can always access the tablet interface on the table in front of them, participants complete trials while sitting in a chair that does not swivel, using the controllers to move within the VR world. In addition to a first-person view of the virtual world, a minimap is displayed to the user. The minimap shows the overhead view of the environment, as well as the locations of the target and player at all times. (The “player” is the participant’s virtual embodiment within the VR environment.) During trials with robot assistance, the minimap also displays the locations of the drones and temporarily displays the locations of any detected people in the environment for 3 s. In the 30×30 unit environment, the drones detect people within a 2×2 unit square area cannot predict the future path of a person, and the drone’s velocity is capped at 30 units per second. To aid in spatial orientation in the environment, the minimap display rotates so that the participant’s view always corresponds to “up” in the minimap. Next to the minimap, the number of lives left and the game time are represented by a bar and time counter. Final game score is the number of treasures collected plus three times the number of lives leftover. Participants began each trial with 8 lives, corresponding to 24 game points; in Fig. 3, we subtract participants’ initial game score from their final game score.

### Tactile interface for user commands

Participants use a TanvasTouch monitor (30, 31) with surface haptics to send commands to the robots. The TanvasTouch renders textures on the smooth screen by modulating friction underneath the user’s fingertip. We create different textures for the borders of the workspace and the user’s location as different textures. The user’s location is represented by a fine texture that results in larger vibrations in the finger. The environment boundaries are represented by a course texture that results in smaller vibrations in the finger. The darkness of a given pixel in Fig. 1A corresponds to the level of friction felt as the participant’s finger brushes over the surface. The display dynamically updates according to the person’s position and orientation to match the minimap visually displayed in the Unity environment. Auditory feedback follows physical interaction with the tablet. The use of a haptic tablet enables the human operator to orient themselves in their VR environment and send commands while wearing the VR headset.

### Shared control of multiple robots

During ergodic control (43–49, 71), the robots use a metric from information theory (72) to minimize the difference between two spatial distributions over the environment: (i) the target distribution representing the expected value of sensory augmentation and (ii) a distribution representing where the robots have visited. One target distribution is provided to all three robots, which autonomously coordinate with each other. During *user coverage control*, the target distribution is provided by the participant shading high-priority regions on a tablet. During *fully autonomous coverage control*, the robots use a computational model of the human’s sensory field and exact locations of previously detected people to autonomously build the target distribution; the robots cannot independently anticipate the participants’ navigation goals. During *shared coverage control*, the human and autonomous distributions are normalized, given equal weight, and combined.

### Procedure

At the beginning of the experiment, 33 participants provided informed consent. The protocol was approved by the Institutional Review Board at Northwestern University. All methods were performed in accordance with the relevant guidelines and regulations. Participants with poor visual acuity without contacts were excluded from this study due to the VR headset. All participants were between ages 18 and 32. After data collection, we excluded participants with <1,000 h of video game experience over their lifetime, as determined by a prestudy questionnaire.

The participants then completed a training session that lasted approximately 1 h and was composed of a tutorial series that familiarized them with the different parts of the experimental setup and interface. After the training session, the researcher placed three ECG electrodes provided by SOMNOmedics (73). The SOMNOtouch PSG device was positioned using a chest strap, and the ${\text{SpO}}_{2}$ soft silicone finger clip was placed on the left hand. Participants performed each of the 10, randomized, 5-min experiment rounds.

### ECG data

ECG measurements are collected using SOMNOtouch RESP throughout the entire experiment. We use somnomedics’s commercial software to compute the “RR interval” for every heartbeat, defined as the time period between consecutive “R” peaks in the ECG signal. Once we remove erroneous measurements, every remaining trial has acceptable data for at least 4 min out of the 5-min trial. Since participant 9’s mean “RR” interval is (>2.9 SDs) lower than the remaining participants mean “RR” interval, we exclude participant 9 from the ECG analysis.

### Optimal agent formulation

The optimal agent formulates its interaction with the environment as a Markov decision process (MDP). An MDP is defined by a set of states (consisting of all intersections in the environment), actions (cardinal directions: north, south, east, and west), transition probabilities (set to equal 1), and rewards (based on game score) (74, 75). The evolution of each state–action pair is determined by simulating a computational model of the environment, determining the next intersection the optimal agent will be at and any resultant changes in reward. A new MDP is built every time the virtual position of the participant arrives at an intersection. The way the environment evolves includes probabilistic components for the adversaries’ movements, which impacts the MDP through the reward. Despite using no discount factor, future rewards regarding the adversaries are attenuated due to increasing uncertainty regarding each adversary’s location. Successive simulations of the optimal agent taking random actions through the environment allows the optimal agent to estimate the expected reward for each state–action pair within the MDP.

Using the MDP for any particular instance where the virtual position of the participant arrives at an intersection, the optimal agent determines the expected reward associated with all four possible actions the participant could take. Expected reward is determined by the Bellman equation (75), looking six intersections into the future. We use the expectation maximization formulation that averages over all possible paths following a particular turn to allow the optimal agent to consider the robustness of any particular turn to future changes in information. The optimal agent chooses the action with the highest expected reward.

### Statistical analysis

For the number of commands and “RR” interval measures, repeated measures ANOVAs with within-participant factors for level of autonomy and building density is performed in R (α=0.05). Assumptions are tested using Shapiro–Wilk test for normality and Mauchly’s sphericity test. To help determine which control paradigm is different from the rest, post hoc, pairwise, and two-way t tests with a Bonferroni correction for multiple comparisons are performed.

For the regret measure, there are an unspecified number of samples per trial. To allow within-trial statistical variation to be considered, we fit the data to a linear mixed model using the LMER function in R with the experimental factors (level of autonomy and building density) as predictors and participant as a random factor. To statistically compare decisions of similar quality, we group decisions according to the maximum impact of the decision on the expected reward (the expected reward of the best decision minus the worst decision). The three groups are [0,1), [1,2), and [2,3), where decisions with a maximum impact of ≥3 are excluded from analyses, and we include the group as another random factor in the linear mixed model. We use Wald $\chi^{2}$ tests to evaluate statistical significance; similar to an ANOVA, the Wald $\chi^{2}$ test evaluates whether a given factor explains some of the variation in an outcome measure. Then, a post hoc Tukey test for multiple comparisons is performed.

For the game score measure, we perform four paired two-sided t tests to compare each of robot-assistance paradigm to the *no robots* condition. For each of the aforementioned tests, we are unable to reject the null hypothesis that the paired difference between paradigms is normally distributed using the Shapiro–Wilk test for normality.

## Supplementary Material

pgae016_Supplementary_Data

## Data Availability

The data that support the findings of this study are available on Zenodo. See Refs. (76), (77), and (78) for the code used to run our experiment. See Refs. (79) and (80) to replicate our data analyses. See Ref. (81) for the annonymized raw data.
