# Supplementary material for: Collaborative robots can augment human cognition in regret-sensitive tasks
Source: PNAS Nexus. 2024 Jan 17;3(2):pgae016. doi: 10.1093/pnasnexus/pgae016 (PMC11079486; doi:10.1093/pnasnexus/pgae016)
Supplement: pgae016_Supplementary_Data [file pgae016_supplementary_data.zip › PNASNEXUS-PNASNEXUS-2023-00973R-s01.pdf]

# Collaborative Robots Can Augment Human Cognition in Regret-Sensitive Tasks

Millicent Schlafly, Ahalya Prabhakar, Katarina Popovic, Geneva Schlafly, Christopher Kim, and Todd D. Murphey

## Supplementary Material

**1 Supplementary Methods** **2**
1.1 Participant Movement in Virtual Reality (VR) . . . 2
1.2 Tactile Interface for User Commands . . . 2
1.3 Experimental Task . . . 3
1.4 Training on the Experimental System . . . 4
1.5 Deriving Several Trajectories From One Interface Curve . . . 5
1.6 Expression of Human's Knowledge . . . 5
1.7 Expression of Autonomy's Knowledge . . . 6
1.8 Shared Knowledge . . . 6
1.9 Ergodic Control . . . 6
1.10 Waypoint Control Paradigm . . . 8
1.11 Simulated Replica of Environment . . . 8
1.12 Relationship to Reinforcement Learning and Partially Observable Markov Decision Processes . . . 9
1.13 Mathematical Representation of Optimal Agent . . . 9
1.14 Metrics Based on the Optimal Agent . . . 12
1.15 Validation of Simulated Replica of Environment . . . 12
1.16 Statistical Analyses for Outcome Measures in the Supporting Information . . . 14
1.17 Included Participants . . . 15

**2 Supplementary Results** **15**
2.1 Supplemental Statistics . . . 15
2.2 Separating Experienced and Novice Participants . . . 16
2.3 Novices, Cognitive Availability Without Robots and Shared Control . . . 17
2.4 Robot Performance at Assisting The Human . . . 19
2.5 Statistical Trends Strengthen in Low-Density Environment . . . 20
2.6 Control Paradigm Preferences and Difficulty Ratings . . . 21
2.7 Simulated Replica of Environment Mirrors Task Performance in Virtual Reality . 23
2.8 Regret Convergence . . . 23

**3 Supplementary References** **27**

## Supplementary Figures

S1 Experimental System Architecture . . . 3
S2 Experimental Task . . . 3
S3 Deriving Several Trajectories From One Input Trajectory . . . 5
S4 Shared Control Paradigm . . . 7
S5 Graphical Representation of a Markov Decision Process . . . 11
S6 The Number of Hours of Video Games Experienced and Novice Participants Played Over Their Lifetime . . . 17
S7 Novices' Cognitive Availability and Decision-Making . . . 18
S8 Human-Robot Team Performance With Novice Participants . . . 19
S9 Effect of Control Paradigm on Robot Behavior . . . 20
S10 Building Layout of High and Low Density Environments . . . 21
S11 Statistical Trends Strengthen in the Low-Density Environment . . . 22
S12 Perceived Difficulty . . . 23
S13 Validation of the Optimal Agent's Representation of the Environment . . . 24
S14 Regret Convergence for the Optimal Agent in the Main Text . . . 25

S15 Regret Convergence for the Optimal Agent with all Task Information . . . . . . . 26

## Supplementary Tables

S1 Interface Interaction Statistics . . . . . . . . . . . . . . . . . . . . . . . . . . . . . . . 16
S2 Cognitive Availability Statistics . . . . . . . . . . . . . . . . . . . . . . . . . . . . . . 16
S3 Decision Regret Statistics . . . . . . . . . . . . . . . . . . . . . . . . . . . . . . . . . . 16
S4 Game Performance Statistics . . . . . . . . . . . . . . . . . . . . . . . . . . . . . . . . 17

# 1 Supplementary Methods

In this section, we provide supplemental details about the experimental platform, control paradigms, optimal agent, study procedures, and data analyses. We design our human subject study to adhere to methodological research standards [S1], including randomization to account for known and unknown confounding factors (i.e., learning or fatigue), controlling random error by using the same room, experimental task/interface, training procedure, and study administrator throughout experiment, and obtaining informed consent from all participants. Although it is not possible to blind either the participants or the research administrator from the control paradigm being used for a given trial, the research administrators refrained from expressing hypotheses about the various experimental conditions, and participants provided subjective feedback on a paper out of the research administrator's view. This study was approved by the Institutional Review Board at Northwestern University.

To support the replication of our experiment or data analyses, all of our code is open source. See [S2], [S3], and [S4] for the code used to run our experiment. See [S5] and [S6] to replicate our data analyses. All raw, anonymized experimental data has been extracted from the source files (either rosbags, electrocardiogram measurement device, or written feedback) using the open-source code and made available to the public in .csv files here [S7]. [S5] also contains annotated statistical output files, a plots for each participant/measure, and various aggregated plots. Please refer to the README.md files for each code repository for further information.

## 1.1 Participant Movement in Virtual Reality (VR)

Walking movement is initiated when controller's trigger button is held with the participant's index finger, and the direction of the forward progress is determined based on player's head orientation, as measured by the VR headset. Users could either gradually change direction of motion by turning their head in a desired direction, or perform 30 degree snap turns by pressing buttons on either side of the controller. When the controller is in the left hand, the left side grip button initiates a 30 degrees counterclockwise rotation and right side grip button initiates a 30 degrees clockwise rotation; rotation buttons are swapped when the controller is held in the right hand. The controller is held with the participant's non-dominant hand so that the participant's dominant hand is available for interaction with the tablet interface. Both the speed of movement as well as the choice and amount of snap rotation is capped to limit the potential side effect of VR motion sickness.

## 1.2 Tactile Interface for User Commands

Using the TanvasTouch [S8, S9], the user sends robot commands by double-tapping the screen, tracing the desired trajectory for each robot (*waypoint control*) or shading the regions of exploratory interest (coverage control paradigms), and double-tapping the screen to complete the input. At the second double-tap, the coordinates are transformed and sent to each robot listening to the commands. *Waypoint control* requires an additional input to indicate which robot to send the command to. Auditory feedback follows physical interaction with the tablet. This is necessary as the headset required for the virtual reality interface obscures the participant's view of the tablet, as shown in Figure S1. Furthermore, it enables participants to maintain their gaze on the surrounding environment. See [S3] for the open-source code.

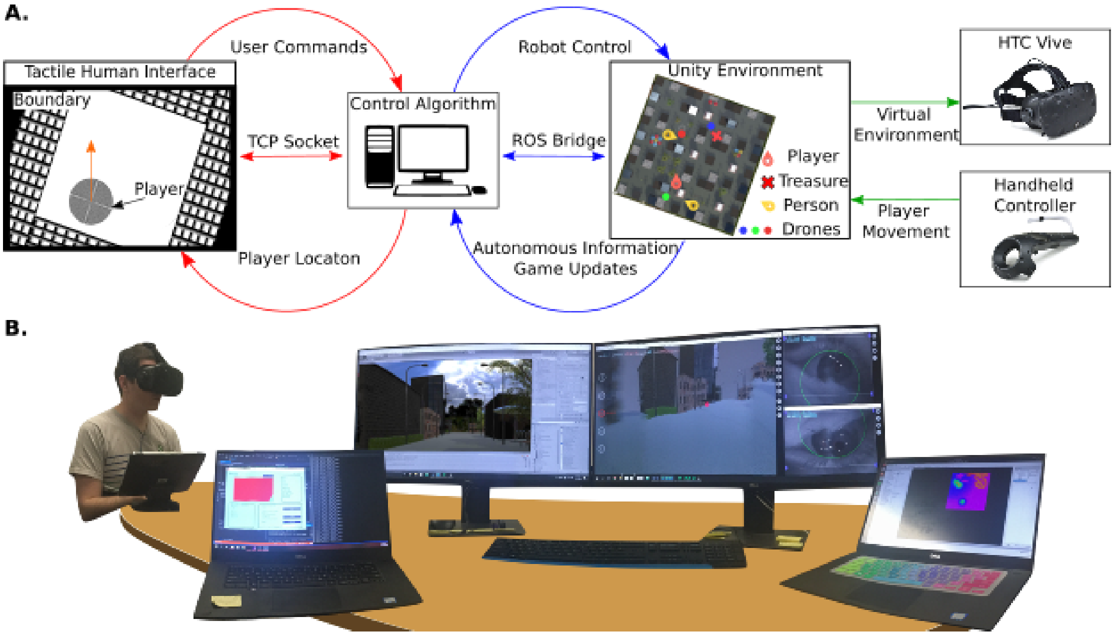


**Figure S1**: **Experimental System Architecture.** Three computers for the tactile human interface, robot control algorithms, and virtual reality environment run our experimental software. Communication between the computers occurs over websockets indicated by colored arrows in (**A**). The minimap in the Unity environment and TanvasTouch world representation rotate together so that forward in the environment is up on both displays. Participants navigated in the environment with the HTC Vive and one handheld controller. (**B**) Participants are seated in chair during the experiment to avoid motion sickness and ensure they can always reach the tactile interface.

### 1.3 Experimental Task

The participants played a virtual reality game in a 30x30 unit grid space with two goals: (1) collect as many treasures as possible and (2) avoid being caught by an adversary, resulting in a lost life. At the end of the five-minute game, the player was given three points for every life leftover and one point for every treasure collected.

The position of the treasure appears as a red "X" on the minimap view in Figure S2. When the participant retrieves the treasure by reaching its location, the next treasure appears both in the environment and on the minimap. While placement is random, there is a constraint of at least 25 units between consecutive treasure locations.

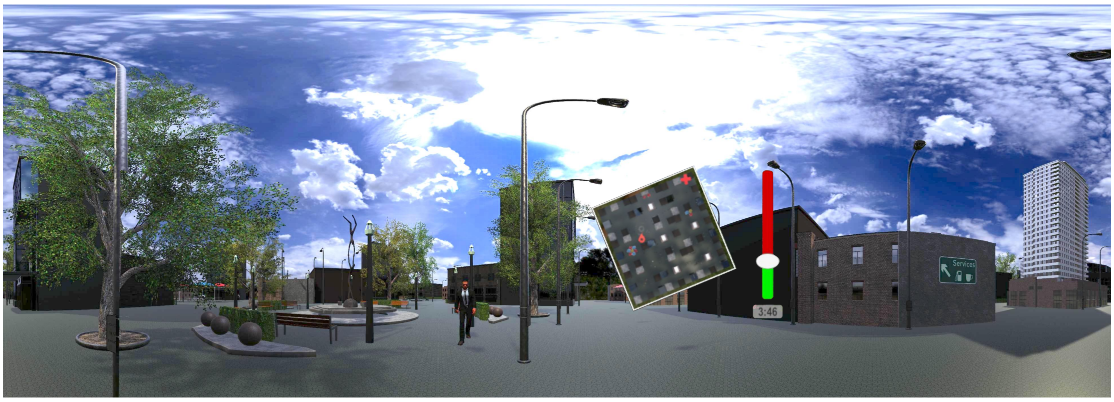


**Figure S2**: **Experimental Task.** Participants perform a virtual reality task that involves collecting treasures and avoiding adversaries. Participants can view an aerial minimap of the environment, game time, and the number of lives remaining.

There are a total of six humanoid characters in the virtual world that patrol based on predefined paths, three pedestrians and three adversaries. When an adversary spots the player, the adversary leaves their patrol route and starts chasing the player. Adversaries can "see" 10 units in front of themselves and 45 degrees in each direction. The adversary chases the player until they (1) "lose" the player (i.e., the adversary had chased the player for 10 units since the last sighting of the player) or (2) "catches" the player, causing the player to lose a life. Following (1), the adversary will stop chasing the player and return to a random point on its patrol route; following (2), the adversary will reappear in a new, random location on its patrol route at least 15 units away from the player, thus, unable to see the player. Because adversaries can perfectly compute the optimal next position during a chase (by choosing the position that minimizes the distance to the player), the speed of each adversary is set to approximately 75% the speed of the player.

As the participants play the game, the robots assist by alerting the player of other people in the environment. We assume that the robots have perfect object recognition capabilities from the camera feedback, such that it can unmistakably recognize items of interest (i.e., humans); however, the object identification cannot distinguish the adversaries from the non-adversarial pedestrians. Once a person has been located, the player then has the opportunity to incorporate this knowledge and reason about the possible adversary location as they transverse the environment. See [S2] for the open-source code.

### 1.4 Training on the Experimental System

When participants arrived for the experiment, they were first reminded what participation would look like, explained the overarching goal of the experiment (to study how a human and robots can best collaborate at a task), and asked to sign a consent form approved by the Institutional Review Board at Northwestern University. Following consent, we asked participants for some basic information about themselves (age, gender, dominant limb, etc.) and asked them to estimate the number of hours spent playing video games over their lifetime. Around 15 minutes into the experiment, we began training. The purpose of the training was to (1) teach participants how to use each system component, (2) familiarize participants with the experimental task, and (3) allow participants to establish game strategies and minimize the effect of learning on our experimental results.

Training began with the virtual reality (VR) environment. The first VR environment shown to participants (low-density or high-density) was randomly selected prior to the participant's arrival. We alternated between environments for the rest of the training protocol. Participants are shown how to navigate the environment using the controllers and explained the rules of the game. They are asked to practice following a path in the environment displayed on the minimap. Then, we displayed a new path and recorded how many times the participant veered from the path. All participants made $\leq 1$ incorrect turn, and were quickly able to return to the path. Next, we asked participants to practice a full five minute game in the VR environment with no robot assistance. In preliminary experiments, we observed that participants were more focused on collecting treasure than avoiding adversaries. To motivate participants to use the robots for avoiding adversaries, we told participants that each life leftover is worth three points and each treasure is worth one point.

Following VR training, participants were explained how to operate the touch interface to provide "path" (*waypoint control*) and "shading" (coverage control paradigms) commands and what auditory feedback will be provided. Participants were instructed to practice double-tapping the tablet screen and providing different types of commands. The taps must occur at the correct frequency or they will not be detected by the interface. Furthermore, participants adjusted to receiving auditory (as opposed to visual) feedback following actions on a screen interface. Participants' ability to operate the touch interface is evaluated by asking participants to provide three "shading" commands and three "path" commands, one to each robot. The number of additional taps required to successfully send the command was recorded; participants required between 0-29 additional taps to send six total commands. We did not exclude any participants due to poor performance on the touch interface evaluation.

The next training section involved the haptic interface. We explained to participants what textures would be rendered during the experiment and how participants can use the rendered textures to orient themselves in the environment. Participants practiced feeling the tablet with

visual aid and an unrotated map as well as without visual aid and a rotated map. For the evaluation, we presented a new haptic arrangement (with a different environment orientation and player position) and asked participants to locate themselves and the border of the environment. All participants completed the request successfully.

Finally, participants completed the full five minute experimental game for the *waypoint control* paradigm and the *user coverage control* paradigm. The order was randomly chosen prior to the participant's arrival. So that participants can observe the effect "shading" commands have on the robots' movements for the coverage control trials, participants practiced with *user coverage control* as opposed to *shared coverage control*. During the trials, we would ask the participant to verbally explain what they were trying to do to ensure the participants' actions matched their intentions and catch misunderstandings. Likewise, participants were encouraged to ask questions. We explained to participants that the robots can improve game performance by locating possible adversaries and that it might be more useful to know that an adversary is one location of the environment as opposed to another, but we did not provide any strategic guidance on where or how often the participants should provide commands to the robots to achieve the best game score possible. The entire training lasted approximately one hour.

### 1.5 Deriving Several Trajectories From One Interface Curve

An arbitrary number of trajectories can be derived from one input curve on the tablet interface, as shown in Figure S3. This is accomplished by using the Dirac delta function and Fourier analyses to map between trajectories (of either the user input or robots) and spatial statistics across the environment. The spatial statistics of the input trajectory serve as the control objective and the spatial statistics of the collective robot trajectories measure the robots' performance at achieving the objective. Using ergodic control [S10–S17], the control algorithm selected for coverage control trials, each robot aims to minimize the difference between the Fourier coefficients representing the input trajectory and the Fourier coefficients representing where the robots have visited.

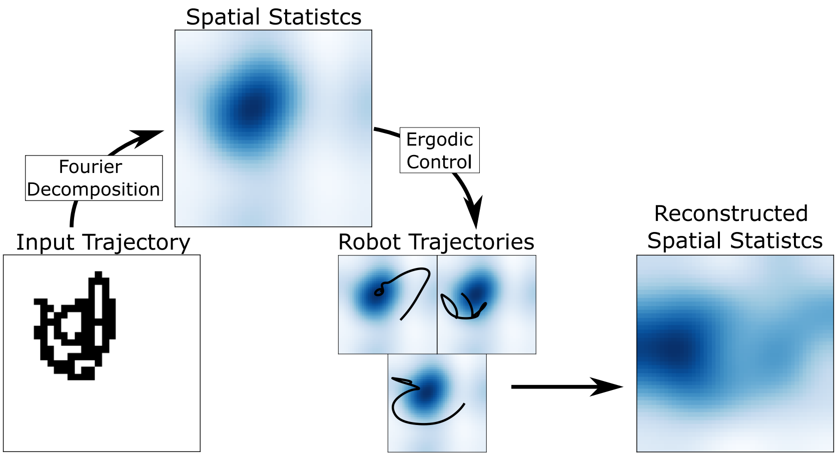


**Figure S3**: **Deriving Several Trajectories From One Input Trajectory.** The input trajectory becomes a distribution using the Dirac delta function, followed by a Fourier decomposition to produce the input trajectory's spatial statistics. Then, we use ergodic control to create three robot trajectories that together recreate the original spatial statistics of the input trajectory.

### 1.6 Expression of Human's Knowledge

By shading the haptic tablet, the human indicates which locations in the environment are high-priority based on knowledge about the environment, intended task, and potential risks. This subsection describes how we obtain a distribution expressing this human knowledge from the set of desired locations that the user wants the robots to prioritize. The locations on the tablet in $\mathbb{R}^2$ touched by the user correspond to locations in the task workspace in the environment, defined as the bounded domain $\mathcal{X}_w \subset \mathbb{R}^2$. The user infers the relationship between the locations on the tablet and $\mathcal{X}_w$ by feeling the tactile features in combination with viewing the minimap

of the environment. Next, we use an invertible function $f : \mathbb{R}^w \to \mathbb{R}^v$ to scale the locations in the task workspace to a box of size $[0,1]^2$ and simplify the ergodic control implementation. The high-priority locations are now represented in another bounded domain $\mathcal{X}_v \subseteq [0,1]^2 \in \mathbb{R}^2$.

Points in $\mathcal{X}_v$ are expressed as a spatial distribution $\phi_u(s)$ where $s \in \mathcal{X}_v$ indicates the position in $\mathcal{X}_v$. We discretize the distribution as a 30x30 grid. Each location identified by the user, rounded to the nearest position $s$ in the 30x30 grid, is assigned the highest priority value of one. The remaining locations in $\mathcal{X}_v$ are assigned a value of 0.005 so that the robots generate minimal coverage over the rest of the workspace. $\phi_u(s)$ is then then normalized such that $\sum_s \phi_u(s) = 1$ and represented using five Fourier coefficients in each exploratory dimension. At the beginning of the *user coverage control* experimental trials, the robots generate a uniform coverage over the workspace (by specifying a uniform initial spatial distribution, $\phi_u(\cdot) = c$ for some $c \in \mathbb{R}$) and updates $\phi_u(s)$ when the user provides a new command. See [S4] for the open-source code.

## 1.7 Expression of Autonomy's Knowledge

When specifying autonomy's knowledge shown in Figure S4, we quantify two main goals: (1) maintaining coverage around the operator, covering blind spots, and (2) reallocating priority to regions around task-relevant objects following discovery. In this work, we assume a predefined knowledge of the environment, such that, given an operator's location $h$, an internal visual coverage model generates a domain $\mathcal{B} \in \mathcal{X}_v$ that represents the operator's blind spots within a local radius. Within the domain $\mathcal{B}$, a distribution $\phi_b(s)$ is created in which all locations where $\|h - s\| < 0.1$ are given a high-importance weight of 10, all locations $\|h - s\| > 0.56$ are given a low-importance weight of 1, and values within the annulus are given an importance weight of $\phi_b(s) = -0.024\|h - s\| + 0.4$ that decreases linearly as one gets further from the operator. All values $s$ in the $[0,1]^2$ grid outside of domain $\mathcal{B}$ are given an importance weight of $\phi_b(s) = 0$. $\phi_b(s)$ is then normalized such that the $\sum_s \phi_o(s) = 1$.

When the locations of task-relevant items of interest are discovered, they are communicated to each robot in the network. An evolving dictionary $\mathcal{D} = \{(p_i, w_i, \Sigma_i)\}_{i=0}^N$ of all the $N$ task-relevant objects is constructed where $p_i$, $w_i$, and $\Sigma_i$ represent the location, importance weight, and variance of the $i^{\text{th}}$ object, respectively. The autonomy's knowledge $\phi_a(s)$ is generated by parameterizing the distribution as a multimodal sum of Gaussians and combining it with the visual coverage model: $\phi_a(s) = 2\phi_b(s) + \frac{1}{\eta}\sum_{\mathcal{D}} \Psi(p_i, w_i)(x)$ where $\eta$ is a normalization factor, and $\Psi(p_i, w_i)(s) = w_i \exp\left(-\frac{1}{2}\|s - p_i\|^2_{\Sigma_i^{-1}}\right)$ for all $s$ in which $\|s - p_i\| < \Pi$. The parameter $\Pi$ is the width of the region of attraction that can be tuned based on the size of the task space and the desired granularity. In this study, we used $\Pi = 0.167$, $\Sigma = \text{diag}(0.067, 0.067)$, and weights $w = 1$ for all items of interest in this work (in $[0,1]^2$ workspace coordinates). This representation generates high importance regions over the operator and any other objects of interest when discovered by the autonomy. Because these items of interests may move over the course of the task, the list of items of interest are constantly updating and after a period of time (15 seconds), the item is removed from the list until located again. The resulting distribution is then normalized such that the $\sum_s \phi_a(s) = 1$ and represented using five Fourier coefficients in each exploratory dimension. See [S4] for the open-source code.

## 1.8 Shared Knowledge

To formally represent shared knowledge, we define a target distribution $\phi(s) = w_u\phi_u(s) + w_a\phi_a(s)$ as a linear combination of the two spatial distributions, the user distribution $\phi_u(s)$ and the autonomous distribution $\phi_a(s)$, where $w_u$ and $w_a$ represent the weights of the individual components of *shared coverage control*, user commands and task specification, respectively. We used weights $w_u = 1$ and $w_a = 1$, giving equal contribution for the shared distribution. The resulting combined distribution is normalized such that the $\sum_s \phi(s) = 1$ and represented using five Fourier coefficients in each exploratory dimension. The *shared coverage control* paradigm is shown in Figure S4. See [S4] for the open-source code.

## 1.9 Ergodic Control

Like prior work [S10–S15], we use ergodicity to compare the temporal statistics of a trajectory of an autonomous system to a desired spatial distribution. In order to define ergodicity and the

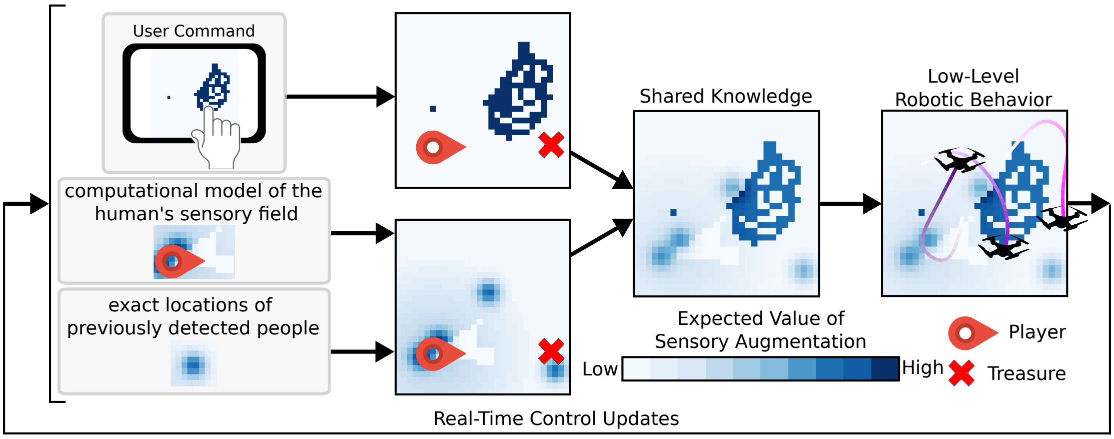


**Figure S4**: **Shared Control Paradigm.** During *shared coverage control*, each robot linearly combines distributions separately provided by the human and the robot. The robot can identify the human's blind spots and prioritize regions around possible adversaries, but does not know the location of the treasure. The robots spend more time in areas of higher interest. During *user coverage control* and *fully autonomous coverage control*, the robots only rely on the distribution provided by the user and the robots, respectively.

ergodic specification that we use, let us assume that the state of a single robot at time $t$ is given by $x(t) : \mathbb{R}^+ \to \mathbb{R}^n$ and the controls to the robot at time $t$ are defined as $u(t) : \mathbb{R}^+ \to \mathbb{R}^m$. The dynamics of the robot are then defined to be the control-affine dynamical system of the form

$$\dot{x}(t) = f(x(t), u(t)) = g(x(t)) + h(x(t))u(t)$$

where $g(x) : \mathbb{R}^n \to \mathbb{R}^n$ is the free, unactuated dynamics of the robot, and $h(x) : \mathbb{R}^n \to \mathbb{R}^{n\times m}$ is the control vector field multiplying the input $u(t)$. Let us now define the robot's time-averaged statistics $c(s, x(t))$ for a trajectory $x(t)$ (i.e., the statistics describing where the robot spends most of its time) for some time interval $t \in [t_i, t_i + T]$ as

$$c(s, x(t)) = \frac{1}{T}\int_{t_i}^{t_i+T} \delta(s - x_v(t))dt,$$

where $\delta$ is the Dirac delta function, $T \in \mathbb{R}^+$ is the time horizon, $t_i \in \mathbb{R}^+$ is the $i^{\text{th}}$ sampling time, $s$ is a point in the exploration space $S^v$, and $x_v(t) \in S^v \cap \mathbb{R}^n$ is the state that intersects with the exploration space, where $v \leq n$. An ergodic metric [S10] that relates the time-averaged distribution $c(s, x(t))$ and an arbitrary spatial distribution $\phi(s)$ is:

$$\begin{aligned}\mathcal{E}(x(t)) &= q \sum_{k\in\mathbb{N}^v} \Lambda_k \left(c_k - \phi_k\right)^2 \\ &= q \sum_{k\in\mathbb{N}^v} \left(\frac{1}{T}\int_{t_i}^{t_i+T} F_k(x(t))dt - \phi_k\right)^2\end{aligned}$$

where

$$\phi_k = \int_{\mathcal{X}_v} \phi(s)F_k(s)ds,$$

$q \in \mathbb{R}^+$ is a scalar weight on the metric, and $c_k, \phi_k$ are the Fourier decompositions (the cosine basis function is used, however, any choice of basis function $F_k$ can be used) of $c(s, x(t))$ and $\phi(s)$ with

$$F_k(x) = \frac{1}{h_k}\prod_{i=1}^{v} \cos\left(\frac{k_i \pi x_i}{L_i}\right)$$

being the cosine basis function for a given coefficient $k \in \mathbb{N}^v$, $h_k$ is a normalization factor defined in, and $\Lambda_k = (1 + \|k\|^2)^{-\frac{v+1}{2}}$ are weights on the frequency coefficients [S10]. A robot whose trajectory $x(t)$ minimizes $\mathcal{E}(x(t))$ as $t \to \infty$ is then said to be optimally ergodic with respect

to the target distribution. That is, the robot spends time in regions of the exploration space proportional to the spatial statistical measure in the exploration space. We use different target distributions for different coverage control paradigms: $\phi(s)$ for *shared coverage control*, $\phi_u(s)$ for *user coverage control*, and $\phi_a(s)$ for *fully autonomous coverage control*. The decentralized ergodic controller used in this experiment relies on barrier functions around $\mathcal{X}_v$ to ensure the robots do not leave the workspace and is adapted from [S14] . $f^{-1}$ maps the robot trajectory to the task workspace $\mathcal{X}_w$. See [S4] for the open-source code.

## 1.10 Waypoint Control Paradigm

For the *waypoint control* method, users specify desired trajectories for each individual robot to follow. To do so, the user double-taps the tactile interface to initiate the command, specifies the desired path for the robot by drawing the line, and double-taps again to indicate the end of the input. Afterwards, the user taps the interface, where the number of taps indicates the robot ID (ID $= 1, 2, ...N$ where $N$ is the total number of robots), to assign the input trajectory to a specific robot. Prior to beginning the task and before learning the starting location of the target and themselves, users are allowed to specify initial paths for each robot. The user could update the desired paths for the robots during the task.

*Waypoint control* was chosen to represent state-of-the-art human-swarm collaboration strategies that enable the human to specify different goals for different robots. It is a simplified version of individually piloting robots (the control strategy used in recent disasters [S18–S21] and representative of current research [S22–S27]). One could integrate *waypoint control* with a leader-follower swarm control strategy [S27–S32] where a group of robots follow the path specified by the human in a formation, similar to [S27], without requiring additional commands from the user. This approach was not pursued here because the size of the virtual environment could only reasonably accommodate three robots. If the three robots followed the same path in a formation, they would not be able to pursue different goals. See [S4] for the open-source code.

## 1.11 Simulated Replica of Environment

We created a simulated replica of the virtual reality (VR) task so that the optimal agent can understand the consequences of its actions. The code governing the behavior of the adversaries and the location of the treasure in response to the participants' actions is written by our research team. The logic is copied from C# to python for simplified use by the optimal agent. The primary structural difference between the simulation and VR task is that we discretize the action space, only allowing the agent to make decisions and change their path at intersections for computational tractability. We determine the participant's decision by looking at the next intersection they arrive at.

Ignoring some low-yield sources of information helps make the problem computationally manageable. When a robot flies over a four unit$^2$ region of the environment and does not see a person, the participant can assign a value of zero to the probability that the region currently contains an adversary. Otherwise, the probability that there is an adversary in the four unit$^2$ region is $\frac{1}{144}$ or smaller (where $\frac{1}{144}$ is the case where no information is currently known about the adversaries and the player is in the high-density environment). Moreover, after a person has been observed, we only simulate its motion for the time it takes for the person to transverse 20 units of the environment, approximately 23.8s. After 23.8s have passed, the person could potentially be located in at least 54% or 48% of locations that are not occupied by buildings in the high-density environment and low-density environment respectively. Through observing participants performing the cognitively demanding task, we conclude that it is unlikely that participants are incorporating the complicated and low-value sources of information described in this paragraph into their game strategy.

We make a few other important simulation design decisions to facilitate the most accurate representation of the task from the participant's perspective. (1) During the VR task, each adversary follows a deterministic patrol route (unless they were chasing the participant or returning to their patrol route). As we are confident that participants did not memorize the adversaries' complicated patrol routes, we decided the adversaries' future nominal movement is best represented by a random walk, where there is an equal probability of the adversary going right, left, or straight at each intersection. (2) We assume that the participant can see 90 degrees in each direction.

Lastly, we made a minor adjustment to how the agent's reward is calculated. In the VR task, the participant only gains in game score when they obtain the treasure. However, reaching a treasure usually takes longer than 60s, which is longer than any reasonable horizon for the optimal agent. Instead, we provide the optimal agent the following continuous reward, where any new treasure is placed at least 25 units away from the participant: reward $= -3 *$ (lives lost) + (change in distance to treasure)/25. If the treasure is found during the simulation, the optimal agent uses the same reward as the player: reward $= -3*$(lives lost)+ $1*$number of treasures found. After a treasure is found, a new treasure is placed in the environment. However, until the instant the new treasure is placed, the optimal agent does not know the location of the treasure and cannot incorporate the new treasure into its path. Regardless, the participants' reward is predominantly determined by their behavior in relation to adversaries. The final 30s of each trial is excluded from all optimal agent analyses. We describe how we validate our simulated replica of the environment later.

## 1.12 Relationship to Reinforcement Learning and Partially Observable Markov Decision Processes

The technical methods supporting the optimal agent's decisions also support two active research fields: reinforcement learning (RL) and partially observable Markov decision processes (POMDPs). Notably, both rely on Markov decision processes (MDPs) to model the world, consisting of a set of agent states, actions, and corresponding rewards. For a Markov decision process to satisfy the Markov property, its evolution into the future must depend only on the present state and must not depend on past history. However, in many applications, particularly those with an agent in an environment, there exists uncertainty in how the environment or the agent within the environment evolves. The RL and POMDP communities take two different approaches to representing uncertainty while attempting to satisfy the Markov property.

In RL [S33], researchers restrict aspects of the world that are considered part of the state to exclude elements that might violate the Markov property. In a typical RL problem with an agent in an environment, uncertainty arises from the environment (i.e., the friction coefficient with the floor), so everything in the environment and the agent's interaction with the environment is excluded from the state. Uncertainty in the world is incorporated into the MDP through the reward.

In POMDPs [S34], researchers use an expansive representation of the state, including hidden aspects of the world as needed to satisfy the Markov property. The state is considered "partially observable" and one updates a probabilistic representation of the state based on observations. A motivating example for POMDPs is human movement—although one can usually include the position of the human in the state, the human's next state depends on the human's intentions and the neurological synapses expressing those intentions, which are typically not observable. In POMDPs, uncertainty in the world is incorporated into the MDP through the state.

In our VR experimental task, there is uncertainty in how the adversaries' position and orientation evolve over time. When the adversary is not chasing the player, at every intersection we assume that there is an equal probability that the adversary could turn right, turn left, or go straight. In RL, the adversaries' behavior would be considered to be a stochastic component of the environment, influencing the evolution of the MDP through the reward. In a POMDP formulation, the adversaries' position would be considered a partially observable component of the state, where observations allow the optimal agent to be more certain about the adversaries' locations. Instead of committing to either the RL or POMDP approach to the problem, we rely on the original problem formulation that inspired both fields, MDPs and the Bellman equation. The implementations of the RL and POMDP approaches would be algorithmically identical—the only difference would be whether the class associated with the adversaries' movement is labeled as part of the "state" or the "environment". Although there have been many recent advances in both RL and POMDPs, relying on the most well-established and widely-verified method available increases our confidence in our results.

## 1.13 Mathematical Representation of Optimal Agent

The optimal agent's understanding of the environment is represented using a Markov decision process (MDP), and the optimal agent's decision-making strategy is determined by the Bellman

**Algorithm 1:** Simulating the Optimal Agent's Actions. This algorithm is repeated 10,000 times every time the participant arrives at an intersection.

```
Input: Game state from human study
Output: Change in reward, the optimal agent's path
Initialize the optimal agent's state as the state of the player (the participant's virtual
  embodiment)
for each robot observation do
    Randomly select whether to include the adversary in simulation, weighted by the
      probability that the robot's observation correctly detected an adversary
    If included, simulate the adversary's movement from the time of observation to the
      current game time
for 6 actions do
    Randomly select the optimal agent's action
    while optimal agent is moving between intersections do
        Move player according to randomly selected action
        for all included adversaries do
            if adversary has recently seen the optimal agent then
                Move adversary to chase the optimal agent
            else
                if adversary is at intersection then
                    Randomly select the next adversary action
                else
                    Move adversary according to current action
            Determine if the adversary has caught the optimal agent
            Determine if the adversary sees the optimal agent
        Determine if the optimal agent sees any of the adversaries
        Determine if the optimal agent has found the treasure
        if the treasure is found then
            Remove treasure from simulation
Compute total reward using the number of times an adversary caught the player and
  the player found the treasure
```

equation. MDPs $\mathcal{M} = \{\mathcal{S}, \mathcal{A}, \mathcal{R}_{sa}\}$ are defined as a set of states $\mathcal{S}$, actions $\mathcal{A}$, and associated rewards for each state-action pair $\mathcal{R}_{sa}$ shown in Figure S5. In other formulations of Markov decision processes, a set of transition probabilities represent uncertainty in the effect of actions on the state; here all transition probabilities are 1 and are ignored in this formulation. The optimal agent's state $s \in \mathcal{S}$ is one of 36 intersections in the environment where $\mathcal{S} = \{I_i\}_{i=1}^{36}$. Depending on one's perspective, as explained in the section on the relationship to reinforcement learning and partially observable MDPs, the state of the adversaries (described in the next paragraph) could be considered as part of the optimal agent's state. The optimal agent can take four actions $\mathcal{A} = \{\text{North}, \text{East}, \text{South}, \text{West}\}$. We find the reward $\mathcal{R}_{sa} \in \mathbb{R}$ for each MDP node by simulating the environment in Algorithm 1.

Every MDP created in this work is specific to a particular game state $\mathcal{G} \in \{\mathbf{p}, \mathbf{g}, \mathcal{H}\}$, where $\mathbf{p} \subseteq [0, 15)^2 \in \mathbb{R}^2$ is the position of the player, $\mathbf{g} \subseteq [0, 15)^2 \in \mathbb{R}^2$ is the position of the treasure, and $\mathcal{H} \subseteq \{H_i\}_{i=0}^{6}$ is a list of the people that the player has recently seen, either using their own vision or the robots. $H \in \{i, \mathbf{x}, a, p, c, t_o\}$ where $i\{i_j\}_{j=0}^{6}$ is the adversary's unique numerical identifier, $\mathbf{x} \subseteq [0, 15)^2 \in \mathbb{R}^2$ is the adversary's position, $a \in \{\text{North}, \text{East}, \text{South}, \text{West}\}$ is the action the adversary is current taking, $c$ is a boolean for whether the adversary is currently chasing the participant, and $t_o \in \mathbb{R}^+$ is the trial time in which the adversary is observed. Every time the participant arrives at intersection in the environment, we have the optimal agent destroy and rebuild a entirely new MDP associated with the new game state.

At each intersection, the optimal agent uses the Bellman equation to determine the value $v$ of following a policy $\pi$ at a state $s \in \mathcal{S}$. A policy describes how the agent will react to

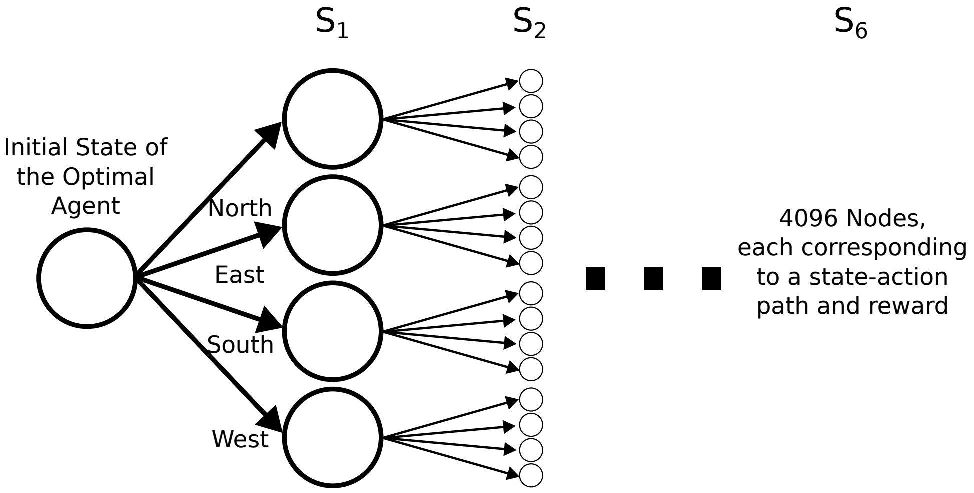


**Figure S5**: **Graphical Representation of a Markov Decision Process.** The initial state of the optimal agent is the position of the participant's virtual embodiment in the virtual reality environment upon arrival at an intersection. The optimal agent simulates various sequences of actions (either North, East, South, or West) through the environment; in total, there are 4096 paths being represented by the Markov decision process. The passing of time is annotated by intersections, where a navigation decision occurs at each of six intersections ($S_1$ - $S_6$) Each circle represents a tuple consisting of the optimal agent's state, action the optimal agent took, and reward.

a future scenario by providing a mapping from the states of the agent and environment to a probability distribution over possible agent actions. We give the optimal agent a conservative future policy where there is equal probability it will take each of the four actions in $\mathcal{A}$, that is $\pi(a_{\text{North}}|s) = \pi(a_{\text{East}}|s) = \pi(a_{\text{South}}|s) = \pi(a_{\text{West}}|s) = 0.25 \quad \forall s \in \mathcal{S}$. We could have chosen a policy that maximizes the expected reward; however, policies that maximize expected reward are susceptible to brittle solutions. In our experiment, new information changes the optimal agent's understanding of the environment, often drastically. Thus, our choice in policy prioritizes paths that provide the optimal agent flexibility to change their mind, possibly due to a newly-detected adversary in the path that previously optimized the expected reward.

The Bellman equation is expressed generally here [S33]:

$$\begin{aligned} v_\pi(s) &= \mathbb{E}_\pi\left[\sum_{k=0}^{\infty} R_{t+k+1}|\mathcal{S}_t = s\right] \\ &= \sum_a \pi(a|s) \sum_{s',r} p(s',r|s,a)[r + \gamma v_\pi(s')] \end{aligned}$$

where $v_\pi(s)$ is the expected reward from following policy $\pi$ at state $s \in \mathcal{S}$, $r \in R$ is the reward for each timestep, $\gamma$ is a discount factor, $t$ is the time at $s$, $s'$ is the state at $t+1$, and $a \in \mathcal{A}$ is the possible actions the agent can take. In the second line, the Bellman equation is expressed as a recursive function where $v_\pi(s)$ depends on $v_\pi(s')$ and $v_\pi(s')$ depends on $v_\pi(s'')$.

Our experimental setup allows us to simplify the more general expression of the Bellman equation. Since our chosen policy $\pi(a|s) = 0.25$ is constant $\forall s \in \mathcal{S}$ and $\sum_a 0.25 = 1$, we can remove the $\pi(a|s)$ term. In our VR setup, the optimal agent taking action $a$ at state $s$ will result in $s'$ with probability $p(s') = 1$, and the virtual embodiment of the optimal agent will enact the action $a$ specified by the optimal agent with probability $p(a) = 1$. The probability of a particular reward will result from a state $p(r|s)$ is evenly distributed across simulations (often referred to as "episodes" in RL). Stochastic aspects of the game are captured in how the environment evolves—if there is a $33.\bar{3}\%$ change that an adversary turns left and causes the optimal agent to lose a life, this scenario will occur in $33.\bar{3}\%$ of simulations. In other uses of the Bellman equation, the agent will simulate some actions in their environment more frequently than others for more computationally-efficient learning. In an effort to only rely on the most widely-verified concepts and methods in the field, the optimal agent follows a policy that executes a random

walk through the environment, meaning that $p(r|s) = \frac{1}{N_{s',r}}$ where $N_{s',r}$ is the number of rewards (each representing one episode) for each state-action node. Gamma is set to 1. Finally, summing over both actions $a$ and the next states $s'$ is redundant. These problem-specific simplifications result in the following equation:

$$v_\pi(s) = \sum_{s',r} \frac{1}{N_{s',r}}[r + v_\pi(s')].$$

In our implementation, we average across the reward at each state-action node in the MDP before solving for the Bellman equation. In this case, there is only one value for the reward for every node and the Bellman equation can be further reduced to:

$$v_\pi(s) = \sum_{s'}[r + v_\pi(s')].$$

We use the above Bellman equation to determine the expected reward of each of the four possible actions at the given state $s$: $v_\pi(s'|a_{\text{North}})$, $v_\pi(s'|a_{\text{East}})$, $v_\pi(s'|a_{\text{South}})$, and $v_\pi(s'|a_{\text{West}})$. The optimal agent takes the action with the highest expected reward and the values for all four actions are used for subsequent analyses.

## 1.14 Metrics Based on the Optimal Agent

Regret is a concept from the reinforcement learning literature and quantifies the difference in reward between the actions executed by an agent (in our case, the human) and the actions an agent would have taken if it were following an alternative, better-performing policy [S35]. The alternative policy is embodied by the optimal agent choosing the action at each intersection with the highest expected reward. Regret $D$ is computed using the following equation:

$$D = \max_{a \in \mathcal{A}}(v_\pi(s'|(a,s))) - v_\pi(s'|(a_{\text{human}}, s))$$

where $\max_{a \in \mathcal{A}}(v_\pi(s'|(a,s)))$ is the expected value from the optimal agent's action. We note that both terms in the regret calculation are based on the same system state, so it is essential to the formulation of regret that the optimal agent has the same information about the environment as the human.

The regret metric is sampled when the participant's virtual embodiment arrives at an intersection. Using playback data from the experiments, we recreate the game state from the perspective of the participant. Algorithm 2 shows our procedure for sampling regret from experimental data. We also record the impact of the decision on expected game reward, quantified by the difference between the largest and smallest expected game reward $\max_{a \in \mathcal{A}}(v_\pi(s'|(a,s))) - \min_{a \in \mathcal{A}}(v_\pi(s'|(a,s)))$.

For the second set of metrics, we use the optimal agent to assess the utility of the robots. We determine (1) whether each of the robots' observations changes the path of the optimal agent and (2) whether the robots' collective behavior, including all new observations, changes the path of the optimal agent in Algorithm 3. Prior work shows that MDPs accurately model how humans make decisions based on available information [S36]. This literature makes a distinction between "model-free" and "model-based" methods, describing habitual and strategic components of behavior respectively [S36, S37]. Since the environmental conditions in every trial change, including the position of the player with respect to the world, the treasure, and the adversaries, we represent human behavior via. the optimal agent as entirely "model-based". If an observation is useful to the optimal agent, we assume it will be useful to the human.

## 1.15 Validation of Simulated Replica of Environment

To validate our model of the environment, the optimal agent completes the task with all information about the adversaries' movements. If our simulated replica of the environment accurately represents the evolving virtual reality game, this optimal agent's prediction of the consequences of participants' actions on game reward will align with the game score received by participants during the experiment. We compute the cumulative regret of the participant compared to the

**Algorithm 2:** Sampling Regret From Experimental Data.

```
Data: Playback data from human subject study
Output: Data file with regret measurements
for each trial do
    Initialize trial data object
    Forward simulate trial data until player reaches an intersection
    Initialize game state with player's position and robot observations
    while trial data remains do
        Initialize Markov decision process (MDP) for game state
        Simulate game using Algorithm 1 10,000 times, populating the MDP (This step
          was parallelized)
        Plug in values for rewards obtained from simulated game data into the Bellman
          equation
        Forward simulate trial data until player reaches the next intersection (This step
          is used to determine which direction the player turned at the prior intersection)
        Save the regret metric and the difference between the largest and smallest
          expected reward
        Update game state with player position and robot observations
        if in the final 30s of trial then
            break
```

optimal agent with all information about the environment. Cumulative regret is $\sum_{i \in \mathcal{I}} D_i$ where $\mathcal{I}$ is a set of the all of intersections the participant passes through until the last 30s of the experimental trial, and $D_i$ is the regret for each intersection. (As a reminder, the optimal agent used for the results in the main text only uses the information available to the participants through their vision or robotic assistance.)

Since the optimal agent has access to all information, we allow it to follow a policy that optimizes the expected reward. The new value associated with each MDP node maximizes over all possible policies the optimal agent could take $v_*(s) = \max_\pi v_\pi(s)$. This requires us to use a modified version of the Bellman equation[S33]:

$$v_*(s) = \max_a \sum_{s',r} p(s', r|s, a)[r + \gamma v_*(s')].$$

By making simplifications specific to our problem statement described in the section on the "Mathematical Representation of Optimal Agent", the Bellman equation becomes:

$$v_*(s) = \max_a [r + v_*(s')].$$

When the optimal agent knows the position of every adversary, it is nearly always able to generate a path through the environment that avoids being caught. The primary way the cumulative regret metric increases is when the optimal agent can not conceive of a way for the participant to avoid being caught by an adversary.

The paths through the environment generated by the optimal agent using the above formulation of the Bellman equation are brittle. This optimal agent does not consider the possibility that it will want to change its path in the future in response to new information about the environment. For the optimal agent that already knows all information about the environment, brittle solutions are acceptable. However, for the optimal agent presented in the main text of the paper that has access to the same information as the participant and expects their understanding of the environment to change in the future, the original Bellman equation that relies on expectation maximization is more appropriate.

**Algorithm 3:** Sampling the Utility of Individual and Collective Robot Observations. For each robot observation, we determine if the observation changes the path of the optimal agent. At each intersection, we determine if all recent observations collectively change the path of the optimal agent.

```
Data: Playback data from human subject study
Output: A data file with the utility of each robot observation and a data file with the
        utility of the collective robot behavior at each intersection
for each trial do
    Initialize trial data object
    Forward simulate trial data until player reaches an intersection
    Initialize game state G_b with player's position and no robot observations
    while trial data remains do
        Determine the optimal agent's next action a_b for G_b by following Algorithm 2
          steps 6-8
        for each observation since last intersection do
            Initialize new game state object G_oi = G_b for current observation
            Update game state G_oi with observation
            Determine the optimal agent's next action a_oi for G_oi by following Algorithm
              2 steps 6-8
            if a_b != a_oi then
                The robot's observation changes the optimal agent's path
            Save the boolean (0 or 1) for whether the robot's observation changes the
              optimal agent's path
        for each observation since last intersection do
            Update game state G_b with observation
        Determine the optimal agent's next action a_c for updated G_b by following
          Algorithm 2 steps 6-8
        if a_b != a_c then
            The collective robot observations change the optimal agent's path
        Save the boolean (0 or 1) for whether the collective robot observations change
          the optimal agent's path
        Update game state with player position
        Obtain list of new robot observations
        if in the final 30s of trial then
            break
```

## 1.16 Statistical Analyses for Outcome Measures in the Supporting Information

We present five outcome measures in the supporting information that are not included in the main text: robot performance at detecting people, utility of individual/collective robot observations, cumulative regret for validating our simulated replica of the environment, and perceived difficulty ratings. Additionally, we include an additional statistical analysis for the "final game score" measure.

For the "robot performance at detecting people" measure, we use a repeated measures ANOVA with within-participant factors for level of autonomy and building density is performed in R ($\alpha = 0.05$). Assumptions are tested using Shapiro-Wilk test for normality and Mauchly's sphericity test. To help determine which control paradigm is different from the rest, post-hoc, pairwise, and two-way t-tests with a Bonferroni correction for multiple comparisons is performed.

"Final game score" is not normally distributed according to the Shapiro-Wilk test for normality. There was an implicit cap on performance due to the authors setting the initial number of lives and the player's speed, thereby restricting the number of targets that could be obtained. We use a generalized linear mixed-effects model using the GLMER function in R and the experimental factors (level of autonomy and building density) as predictors. For game performance,

we chose to parameterize the data using a Poisson distribution because a participant's final score is comprised of the number of times particular events happen during a particular trial (e.g. a life is lost or a target is acquired). We use the same statistical tests for the "cumulative regret" measure as the "final game score" measure.

"The utility of individual/collective robot observation" measures are not normally distributed according to the Shapiro-Wilk test for normality. We fit both measures to a generalized linear mixed-effects model using the GLMER function in R and the experimental factors (level of autonomy and building density) as predictors. Since each sample provides a binary value (1 if the observation changed the optimal agent's path and 0 if it did not), we fit the data to a binomial distribution.

For all linear mixed-effects models, we use Wald Chi-squared tests to evaluate for statistical significance; similar to an ANOVA, the Wald Chi-squared test evaluates whether a given factor explains some of the variation in an outcome measure. Then, post-hoc Tukey tests for multiple comparisons are performed.

The "perceived difficulty rating" measure is not normally distributed according to the Shapiro-Wilk test for normality. In line with standard procedures for handling questionnaires like the Likert scale [S38], we use non-parametric rank tests to determine statistical differences between the control paradigms. We used the Friedman test with blocks for "subject" as an alternative to an ANOVA and the Wilcoxon Signed Rank Sum Test as an alternative to t-tests.

### 1.17 Included Participants

During the experiments, we sometimes experienced technical difficulties that prevented the completion of the experiment. For the number of interface interactions and cognitive availability results, we only included participants with data for all ten trials. Interface interaction results and game score results in the main text of the paper include 31 (24 experienced and 7 novice) participants. Cognitive availability results include 29 (23 experienced and 6 novice) participants. Since Participant 9's mean 'RR' interval is much lower than the remaining participants (Participant 9's mean 'RR' interval is 2.9 standard deviations away from the mean), we exclude Participant 9 from the cognitive availability analysis. Two participants are missing only ECG data for one of the experimental trials.

Since the game score results are not normally distributed, we use a generalized linear mixed-effects model for supplementary statistical tests in the supplementary information. As opposed to repeated measures ANOVAs, generalized linear mixed-effects models allow the inclusion of partial datasets. We are able to include data from two additional participants who completed nine out of the ten trials. Game score results in the supplementary information include 33 (25 experienced and 8 novice) participants. Since we also used linear mixed models for the metrics involving the optimal agent, including decision regret and robot utility, we can include data from participants missing some of the data necessary to playback the entire trial. Results based on the optimal agent include 33 (25 experienced and 8 novice) participants where at least eight of the ten trials were included for each participant.

The survey was added to the experimental protocol after a few participants had already participated in the study. Therefore, perceived difficulty results include 31 (24 experienced and 7 novice) participants. For the number of robot observations metric, we only included the participants 29 (23 experienced and 6 novice) participants with data for all ten trials. One participant voluntarily ended participation due to vision discomfort in the virtual reality environment.

## 2 Supplementary Results

### 2.1 Supplemental Statistics

For every ANOVA and Wald Chi-squared test reported in the main text of the paper, we performed post-hoc t-tests. The results are presented in Tables S1, S2, S3, and S4. While ANOVA and Wald Chi-squared tests evaluate if the metric is affected by the experimental condition, post-hoc statistical tests help determine which of the experimental conditions are different from the others and the direction of the trend.

**Table S1**: **Interface Interaction Statistics For Experienced Participants.** Control paradigm has a statistically significant effect on the number of commands used to operate the interface ($p < 0.001$, $F = 10.21$). We additionally perform paired t-tests with a Bonferroni correction for multiple comparisons (where each p-value is multiplied by three). Statistically significant comparisons ($p < 0.05$) are in bold. CC stands for coverage control.

| | Waypoint | User CC | Shared CC |
|---|---|---|---|
| Waypoint | - | $t(47) = 0.25$, $p = 2.41$ | $\boldsymbol{t(47) = 3.80}$, $\boldsymbol{p = 0.001}$ |
| User CC | - | - | $\boldsymbol{t(47) = 3.54}$, $\boldsymbol{p = 0.003}$ |
| Shared CC | - | - | - |

**Table S2**: **Cognitive Availability Statistics For Experienced Participants.** Control paradigm has a statistically significant effect on mean 'RR' interval ($p < 0.001$, $F = 5.47$). We perform paired t-tests with a Bonferroni correction for multiple comparisons (where each p-value is multiplied by ten). Statistically significant comparisons ($p < 0.05$) are in bold. CC stands for coverage control.

| | No Robots | Waypoint | User CC | Shared CC | Autonomous CC |
|---|---|---|---|---|---|
| No Robots | - | $\boldsymbol{t(45) = 3.19}$, $\boldsymbol{p = 0.025}$ | $t(45) = 0.76$, $p = 4.527$ | $t(45) = 0.63$, $p = 1.000$ | $t(45) = -0.13$, $p = 1.000$ |
| Waypoint | - | - | $t(45) = -2.42$, $p = 0.199$ | $\boldsymbol{t(45) = -3.12}$, $\boldsymbol{p = 0.0317}$ | $\boldsymbol{t(45) = -3.60}$, $\boldsymbol{p = 0.008}$ |
| User CC | - | - | - | $t(45) = -0.11$, $p = 1.000$ | $t(45) = -0.77$, $p = 1.000$ |
| Shared CC | - | - | - | - | $t(45) = -0.73$, $p = 1.000$ |
| Autonomous CC | - | - | - | - | - |

**Table S3**: **Decision Regret Statistics For Experienced Participants.** Control paradigm has a statistically significant effect on decision regret ($p = 0.141$, $\chi^2 = 1.96$). We perform a Tukey test for multiple comparisons. Statistically significant comparisons ($p < 0.05$) are in bold. CC stands for coverage control.

| | No Robots | Waypoint | User CC | Shared CC | Autonomous CC |
|---|---|---|---|---|---|
| No Robots | - | $\boldsymbol{t(6388) = -3.55}$, $\boldsymbol{p = 0.003}$ | $t(6388) = 0.76$, $p = 0.940$ | $t(6388) = 0.328$, $p = 0.997$ | $t(6388) = 2.292$, $p = 0.143$ |
| Waypoint | - | - | $\boldsymbol{t(6388) = 5.80}$, $\boldsymbol{p = 0.049}$ | $\boldsymbol{t(6388) = 5.48}$, $\boldsymbol{p < 0.001}$ | $\boldsymbol{t(6388) = 8.166}$, $\boldsymbol{p < 0.001}$ |
| User CC | - | - | - | $t(6388) = -0.638$, $p = 0.968$ | $t(6388) = 2.12$, $p = 0.207$ |
| Shared CC | - | - | - | - | $\boldsymbol{t(6388) = 2.93}$, $\boldsymbol{p = 0.027}$ |
| Autonomous CC | - | - | - | - | - |

## 2.2 Separating Experienced and Novice Participants

While all participants receive the same training and exposure to the experimental setup, playing video games provides relevant experience to our experimental task. Video game experience may translate to a better understanding of how to maneuver in the environment and reason under pressure, freeing up cognitive availability for strategy. Additionally, many modern video games require players to quickly reason about autonomous characters and uncertain information. Experienced participants may be better prepared to exploit the advantages of each control paradigm. Furthermore, prior work shows that experience affects how humans interact with autonomous systems in other settings [S39].

We measure video game experience by asking participants to estimate the number of video games played over the participant's lifetime. When the data are plotted in Figure S6, it can be observed that there is a natural split around 1000 hours. There are no participants with between 580 and 1000 hours of video game experience. Therefore, we split the participants into two groups: 25 experienced participants with $\geq$ 1000 hours of video game experience and 8 novice participants with $<$ 1000 hours of video game experience.

Justifying our choice to separate experienced and novice participants, we find a significant interaction effect between expertise level (experienced vs. novice) and control paradigm in cognitive availability ($p < 0.001$, $F = 5.81$) and regret ($p < 0.001$, $\chi^2 = 40.33$), as well as a marginally significant interaction effect in the final game score measure described later ($p = 0.089$, $\chi^2 = 8.08$). These statistical results mean that the trends across control paradigms is different in experienced and novice participants. The main text of the paper includes only

**Table S4: Game Performance Statistics For Experienced Participants.** Control paradigm has a statistically significant effect on game score ($p = 0.013$, $\chi^2 = 12.68$). We perform a Tukey test for multiple comparisons. No comparisons are statistically significant ($p < 0.05$). CC stands for coverage control.

| | No Robots | Waypoint | User CC | Shared CC | Autonomous CC |
|---|---|---|---|---|---|
| No Robots | - | $t(244) = 0.33$, $p = 0.997$ | $t(244) = -0.26$, $p = 0.999$ | $t(244) = -2.23$, $p = 0.163$ | $t(244) = -0.68$, $p = 0.961$ |
| Waypoint | - | - | $t(244) = 0.60$, $p = 0.975$ | $t(244) = -2.60$, $p = 0.074$ | $t(244) = -1.02$, $p = 0.847$ |
| User CC | - | - | - | $t(244) = -2.03$, $p = 0.254$ | $t(244) = -0.43$, $p = 0.993$ |
| Shared CC | - | - | - | - | $t(244) = 1.62$, $p = 0.488$ |
| Autonomous CC | - | - | - | - | - |

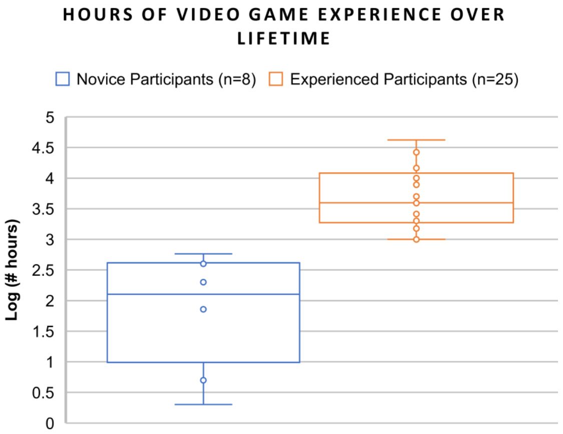


**Figure S6: The Number of Hours of Video Games Experienced and Novice Participants Played Over Their Lifetime.** Participants are separated into two groups—experienced and novice participants—according to the number of hours of video games they played over their lifetime. The cutoff is at 1000 hours ($\log(1000) = 3$), where there is a natural split in the data.

results from experienced participants, but novice results are included here in the supporting information.

## 2.3 Novices, Cognitive Availability Without Robots and Shared Control

Being assisted by robots impacts the cognitive availability of the novices differently than experienced participants. For experienced participants, operating the robots reduces cognitive availability, particularly for *waypoint control*. However, for novices, any robot assistance—regardless of whether it requires the user to provide commands or results in a higher game score—improves cognitive availability. Although the effect of control paradigm on cognitive availability across the six novices is insignificant ($p = 0.141$, $F(4, 20) = 1.96$), post-hoc testing reveals that any statistical trend that does exist can be attributed to the differences in cognitive availability during the *No Robots* condition compared to the remaining conditions where the participant is assisted by robots where the complete statistical results can be found here [S5]. Moreover, there is some evidence that novices' cognitive availability is impacting their decision-making. Although we do not have enough data points for a linear mixed-effects model for the regret metric to converge, Figure S7 shows novices' percent regret under the *No Robots* condition is around 38% and much higher than experienced participants (whose average percent regret is approximately 30%). It is possible that the illusion of task assistance alleviates some of the novices' stress, increasing cognitive availability.

The novice cognitive availability results further suggest that participants who are less familiar with autonomous systems may require additional cognitive resources to reason about shared control paradigms. Unlike experienced participants, novices experience less cognitive availability during the shared control condition compared to the other control paradigms. Told to achieve the best game score possible, novices may be considering what commands would complement the autonomy's knowledge. However, with less video game experience, the process of deliberating could be cognitively taxing for novices. Furthermore, novices do not experience the same benefit in game performance using shared control in Figure S8. Although control paradigm is not a

statistically significant factor among novices ($p = 0.15$, $\chi^2 = 6.80$), game performance of novices is best using fully autonomous robots.

To avoid biasing the results, we made the choice to not provide participants any strategic guidance for how to use *shared coverage control*. Instead, the training, described in Section S6, was primarily focused on how to operate the interface correctly. It is unclear how much novices' cognitive availability and performance using *shared coverage control* would improve with additional practice and guidance. These results are further limited by the small number of novice participants.

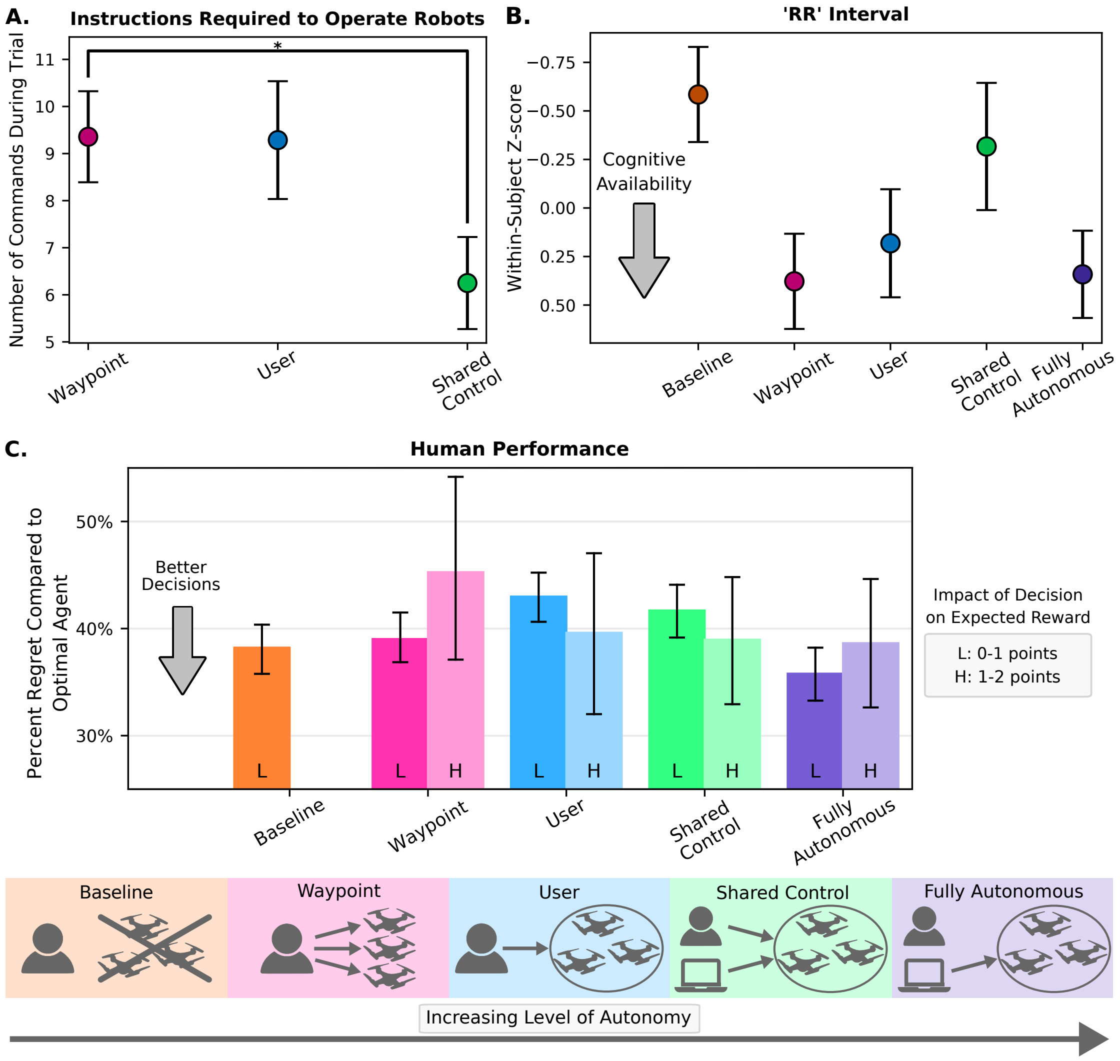


**Figure S7**: **Novices' Cognitive Availability and Decision-Making.** The eight novices with less than 1000 hours of video game experience over their lifetime respond differently to robotic assistance than the experienced participants presented in the main text of the paper. Despite *shared coverage control* requiring fewer instructions (**A**), novices have less cognitive availability using *shared coverage control* than any other condition with robot assistance (**B**). Unlike experienced participants, novices have the least cognitive availability during trials with no Funding acquisition: TDM, AProbotic assistance. Novices' percent regret (**C**) during *No Robots* trials is around 38% and much higher than experienced participants (whose average percent regret is approximately 30%). The repeated measures ANOVAs for the number of commands and 'RR' interval is insignificant. For the human performance results, there is insufficient data to fit a linear mixed-effects model with all necessary factors, so no statistical tests are performed. Error bars indicate standard error. The *No Robots*-H bar is removed because there are only 17 instances where novices encountered a high-regret decision with no robot assistance. Asterisks indicate statistical significance from pairwise t-tests with corrections for multiple comparisons where $^*p \leq 0.05$.

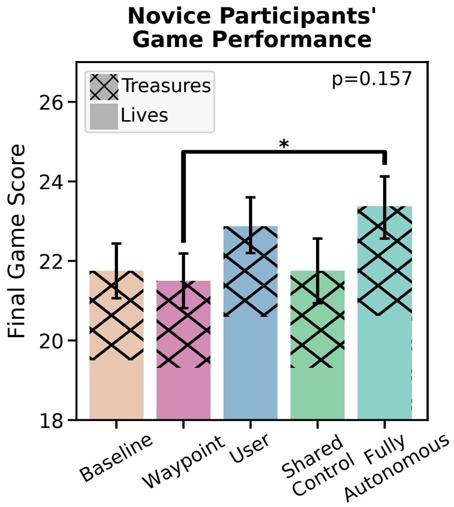


**Figure S8**: **Human-Robot Team Performance With Novice Participants.** Novices obtain the best final game score using fully autonomous robots, although results are insignificant. Final game score is the number of treasures collected plus three times the number of lives leftover. This formula is provided to participants prior to data collection. Error bars indicate standard error. The asterisk indicates statistical significance: $^*p \leq 0.05$.

## 2.4 Robot Performance at Assisting The Human

If the human has a better understanding of their environment, they can better predict the impact of their decisions on game reward. While cognitive availability enables participants to make better decisions based on the information available, more information and better quality information improves the maximum absolute performance we can expect from the human. To assess the quantity of information provided to the participant, we look at the number of people, each a possible adversary, detected during each five minute trial in Figure S9. Level of autonomy has a statistically significant effect on robot performance at detecting people ($p < 0.001$, $F(3, 81) = 104.01$) with the number of people detected increasing as the robots manage more of their own behavior.

To assess the quality of information provided by the robots to the human, we look at the probability that each new observation changes the path of the optimal agent. Many studies show that Markov decision processes, the framework supporting the optimal agent's decisions, accurately model how humans make decisions based on available information [S36], supported by compelling neurological analogies [S40]. If the observation changes the path of the optimal agent, we assume that the observation is useful to the human's current navigation decisions. Level of autonomy has a statistically significant effect on probability that a new observation changes the optimal agent's path ($p = 0.002$, $\chi^2 = 14.91$). At lower levels of autonomy where the human has more control over the robots' behavior, each of the robots' observations are more useful to the human's current navigation decisions.

Both the quantity and quality of information provided by the robots are considered in the last metric we use to assess the robot's performance at assisting the human. We evaluate the probability that all new observations collectively change the path of the optimal agent, considering all robot observations that occur before the participant arrives at the current intersection and after the prior intersection. Level of autonomy does not have a statistically significant effect on probability that the new observations change the optimal agent's path ($p = 0.195$, $\chi^2 = 4.70$). Low statistical power is partially due to the comparatively few samples for assessing the utility of collective robot behavior (one sample per intersection) in contrast to the utility of each robot observation (one sample per robot observation). Although the trend is not statistically significant, we find that the collective robot behavior during *shared coverage control* is most useful to the human.

The *shared coverage control* paradigm balances the expressed coverage goals of the human end-user with the robot's computational understanding of the evolving environment. While the robots discover a greater quantity of people under the *fully autonomous coverage control*

condition, the individual observations are less relevant to the human's real-time needs; this is unsurprising as there is no mechanism for the human to communicate their real-time needs to the autonomy.

The above results include trials from both experienced and novice participants. There is no interaction effect between expertise and control paradigm in the robot performance at detecting people ($p = .681$, $F(3, 81) = 0.504$) or the utility of the collective robot behavior to the human ($p = 0.825$, $\chi^2 = 0.90$). The interaction effect between expertise and control paradigm is statistically significant for the utility of each robot observation to the human ($p = 0.049$, $\chi^2 = 7.85$) due to the novices in the high-density environment experiencing a greater difference between the user and fully autonomous conditions.

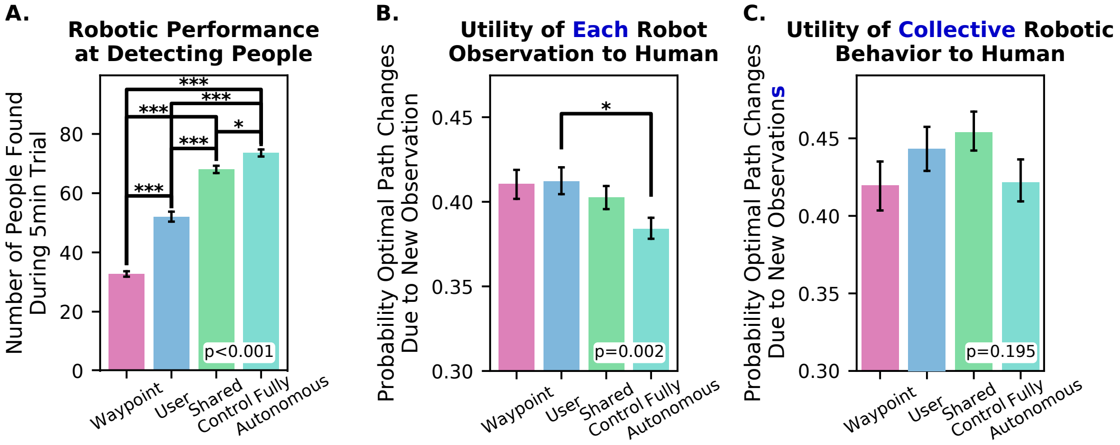


**Figure S9**: **Effect of Control Paradigm on Robot Behavior.** As the control authority assigned to the robots increases, (**A**) the robots are more efficient at augmenting the human's sensory system, discovering more possible adversaries in the environment. (**B**) However, the probability that each individual robot observation will change the optimal agent's decision at the next intersection decreases as the level of autonomy increases. In other words, when the level of autonomy is low, the regions explored by the robots are more relevant to the human's real-time information acquisition needs. (**C**) When all recent observations are considered together, balancing the quantity of information provided by the robots in **A** and the quality of each robot observation in **B**, the utility of the collective robot behavior is highest using *shared coverage control*. We show the probability that optimal agent's path is changed by including all robot observations occurring after the prior intersection. Error bars indicate standard error. Asterisks indicate statistical significance: $^*p \leq 0.05$, $^{**}p \leq 0.01$, and $^{***}p \leq 0.001$.

## 2.5 Statistical Trends Strengthen in Low-Density Environment

Participants complete the task in two virtual reality environments shown in Figure S10 that differ in building density. The high-density environment is designed to emulate several blocks of a city, and one can draw 4 lines of symmetry through the environment (a horizontal, a vertical, and two diagonal lines). The environmental regularity is only broken by the locations of the treasure and adversaries. In the low-density environment, 25% of the buildings are removed and replaced with parks, benches, and statues, adding more spatial variation to the city layout. When the participant is in a region of the environment that has fewer buildings, they can see more of their surroundings with their own vision (aiding in strategic planning), but are more susceptible to being spotted by an adversary. Depending on the circumstances, navigating to a low-density region of the environment could be beneficial or harmful to the game score. The spatial variation of buildings in the low-density environment adds complexity to the path planning task.

In our study, we find that statistical trends relevant to participants' cognitive availability strengthen in the low-density environment compared to the high-density environment in Figure S11. While control paradigm *does not* significantly affect cognitive availability in the high-density environment ($p = 0.256$, $F(4, 88) = 1.36$), control paradigm *does* significantly affect cognitive availability in the low-density environment ($p = 0.001$, $F(4, 88) = 4.80$). For the game

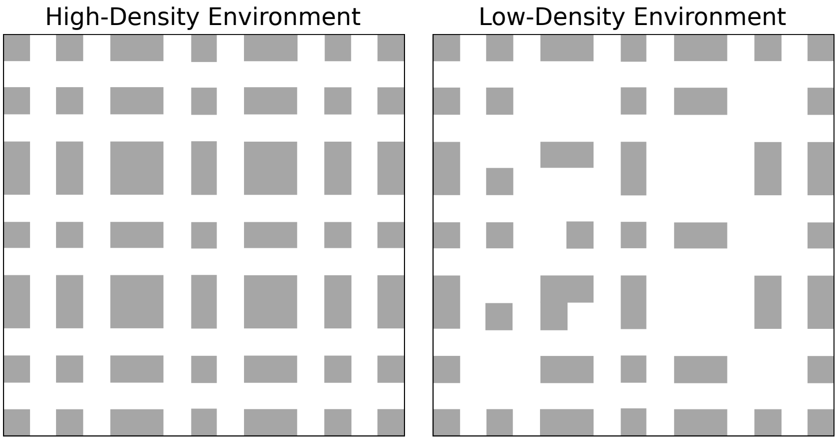


**Figure S10**: **Building Layout of High and Low Density Environments.** The grey blocks are occupied by buildings. One can draw 4 lines of symmetry through the high-density environment (a horizontal, a vertical, and two diagonal lines). In the low-density environment, 25% of the buildings are removed and replaced with parks, benches, and statues, adding more spatial variation to the city layout. When the participant is in a region of the environment that has fewer buildings, they can see more of their surroundings with their own vision (aiding in strategic planning), but are more susceptible to being spotted by an adversary. The spatial variation of buildings in the low-density environment adds complexity to the path planning task.

performance measure, control paradigm *is not* a statistically significant factor in the high-density environment ($p = 0.257$, $\chi^2 = 5.31$), but *is* a statistically significant factor in the low-density environment ($p = 0.026$, $\chi^2 = 11.01$). For the regret measure evaluating human performance, while control paradigm is a statistically significant factor in both the high-density environment ($p < 0.001$, $\chi^2 = 106.92$) and the low-density environment ($p < 0.001$, $\chi^2 = 49.94$), a few paired t-test comparisons between control paradigms in Figure S11 are statistically significant in the low-density environment and are not statistically significant in the high-density environment (the comparison between *No Robots* and user or *No Robots* and *shared coverage control*). This finding confirms prior work that shows that the effect of increased cognitive load on performance is greatest when task demands are high [S41–S43]. It is possible that differences in cognitive availability may not have been discernible with 25 participants and a simpler task. High task complexity may facilitate the discovery of trends in cognitive availability in other contexts.

## 2.6 Control Paradigm Preferences and Difficulty Ratings

After each experimental trial, participants are asked to rate the trial's difficulty. The rating occurs on a sheet of paper out of view of the researcher administering the experiment. The trials are labeled as "Trial 1," "Trial 2,"…"Trial 10" as opposed to the experimental condition. Ratings are between 1 and 10 with 1 indicating the trial is difficult and 10 indicating the trial is easy. Experienced and novice participants are combined in this section and in Figure S12.

Level of autonomy has a statistically significant effect on perceived difficulty in both the high-density environment ($p = 0.006$, $\chi^2 = 14.47$) and the low-density environment ($p = 0.024$, $\chi^2 = 11.28$), with the trial being perceived as easier using higher levels of autonomy. The Wilcoxon test for group difference reveals a statistically significant difference between the perceived difficulty of the *No Robots* condition and *shared coverage control* ($p = 0.049$). In the post-experiment questionnaire, the majority of participants preferred *shared coverage control* (58.6%) compared to *user coverage control* and *fully autonomous coverage control*.

As survey results are frequently used in the robotics community to assess the effect of a particular method on the user, it is worthwhile to reflect on how the perceived difficulty ratings are similar and dissimilar from the cognitive availability results. Both the perceived difficulty and cognitive availability results intend to capture how much effort is required to perform the task under different experimental conditions. Across the control paradigms with robot assistance—*waypoint control* and the three types of coverage control—the trends across experienced participants are similar with *fully autonomous coverage control* requiring the least effort. However, although participants have high cognitive availability during the *No Robots* condition,

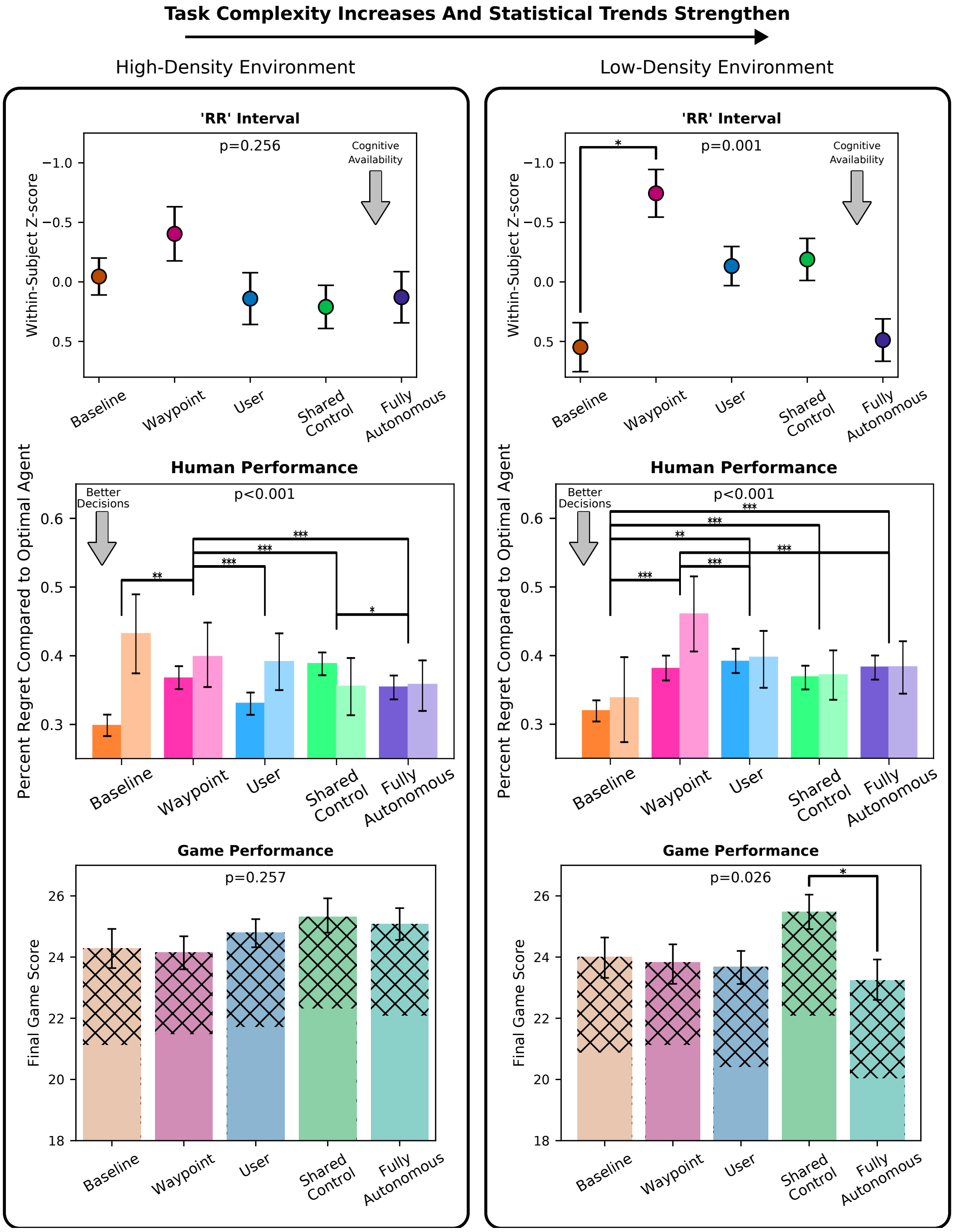


**Figure S11**: **Statistical Trends Strengthen in the Low-Density Environment.** For the 'RR' interval and game performance, level of autonomy is a significant factor for the low-density environment and not a significant factor for the high-density environment. For human performance, more of the pairwise t-tests are significant. Error bars indicate standard error. Asterisks indicate statistical significance: $^{*}p \leq 0.05$, $^{**}p \leq 0.01$, and $^{***}p \leq 0.001$.

participants perceive trials with no robot assistance to be the most difficult. A study comparing manual and autonomous driving found a similar trend [S39]. While the survey results are clouded by how much support the participant feels they are receiving, perceived support only affects cognitive availability as measured using the 'RR' interval if it also affects the participants' physical or cognitive state, possibly by increasing stress. In contrast to the perceived difficulty, the 'RR' interval is heavily influenced by mental workload or the amount of working memory being used. There are many tasks, such as listing prime numbers, that may require large mental resources but are perceived as "easy". While the user's perception of a technology is important

in determining whether it gets adopted, here we are primarily interested in what the human-robot paradigm does to the user's ability to make decisions, which is better captured by cognitive availability measures.

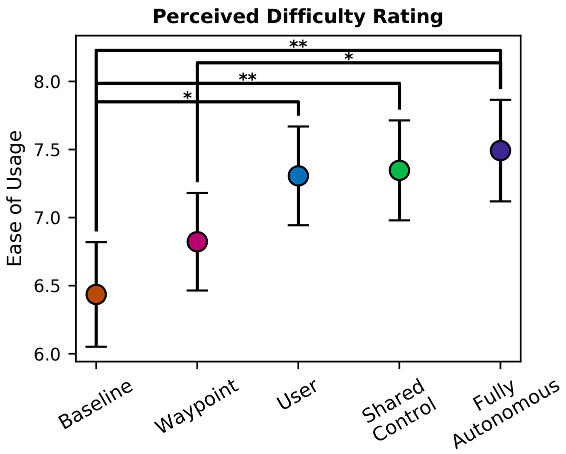


**Figure S12**: **As level of autonomy increases, participants perceive trials as easier.** Participants rated the trial's difficulty, where 1 signifies a more difficult trial and 10 signifies an easier trial. Error bars indicate standard error. The asterisk indicates statistical significance: $^{*}p \leq 0.05$ and $^{**}p \leq 0.01$.

## 2.7 Simulated Replica of Environment Mirrors Task Performance in Virtual Reality

In Figure S13, participants' cumulative regret compared to the optimal agent with all task information mirrors the performance results. Experienced participants' have the least regret using *shared coverage control*, by around one game point. Using a generalized linear mixed model with a Poisson link function, human-robot paradigm has a marginally significant affect on cumulative regret ($p = 0.092$, $\chi^2 = 8.00$). The posthoc pairwise t-tests with a Tukey correction for multiple comparisons show the lowest p-values for comparisons between *shared coverage control* and the other paradigms, indicating that the marginally significant affect of control paradigm on cumulative regret can be attributed to the difference between *shared coverage control* and the remaining control paradigms. We interpret the lack of pairwise statistical trends, particularly among trials that are not *shared coverage control*, to be a reason for increased confidence in our model. Assumptions we made in the creation of the simulated environment do not introduce biases in the regret metric that disproportionately affect one of the experimental conditions.

## 2.8 Regret Convergence

The optimal agent determines the reward of each action by simulating the system into the future for various action sequences, populating a Markov decision process (MDP). Since the adversaries' movement has a stochastic component, one needs multiple simulations for any given action sequence to capture the probability that the agent will get caught by an adversary. The number of agent actions modeled by each MDP is determined by parameter $H$; simulating further into the future improves accuracy but exponentially expands the size of the MDP, increasing computational complexity. The number of simulations is determined by parameter $N$. During each simulation, we randomly select the agent's actions, so there are no guarantees that each of the $4^H$ state-action nodes have been visited. To determine the number of actions and simulations for convergence, we tested a range of values for randomly selected subjects.

In Figures S14 and S15, we plot regret as the parameters $H$ and $N$ increase for randomly selected subjects and intersections. In Figure S14, we look at the optimal agent when it can only access information available to the participant reported in the main text of the paper. In Figure S15, we look at the optimal agent when it has access to all of the adversaries' movements

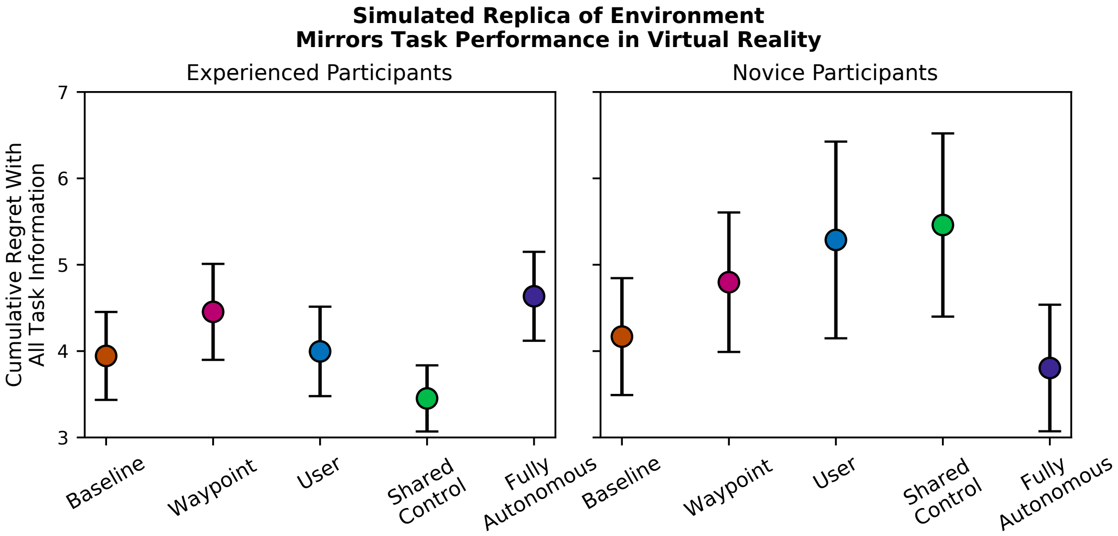


**Figure S13**: **Validation of the Optimal Agent's Representation of the Environment.** Experienced participants have the least cumulative regret using *shared coverage control*. Similarly, experienced participants obtain the highest game score using the *shared coverage control* paradigm. Novice participants have higher cumulative regret than experienced participants, corresponding to worse performance. Novices obtain the highest game score using fully autonomous robot and similarly experience the lowest cumulative regret. These results confirm that our simulated representation of the environment does not introduce unexpected trends in the regret metric.

used for verification in the previous subsection. We determine that six actions and 10,000 simulations are sufficient. In six actions, the agent can transverse the entire environment horizontally or vertically and is typically able to reach the treasure.

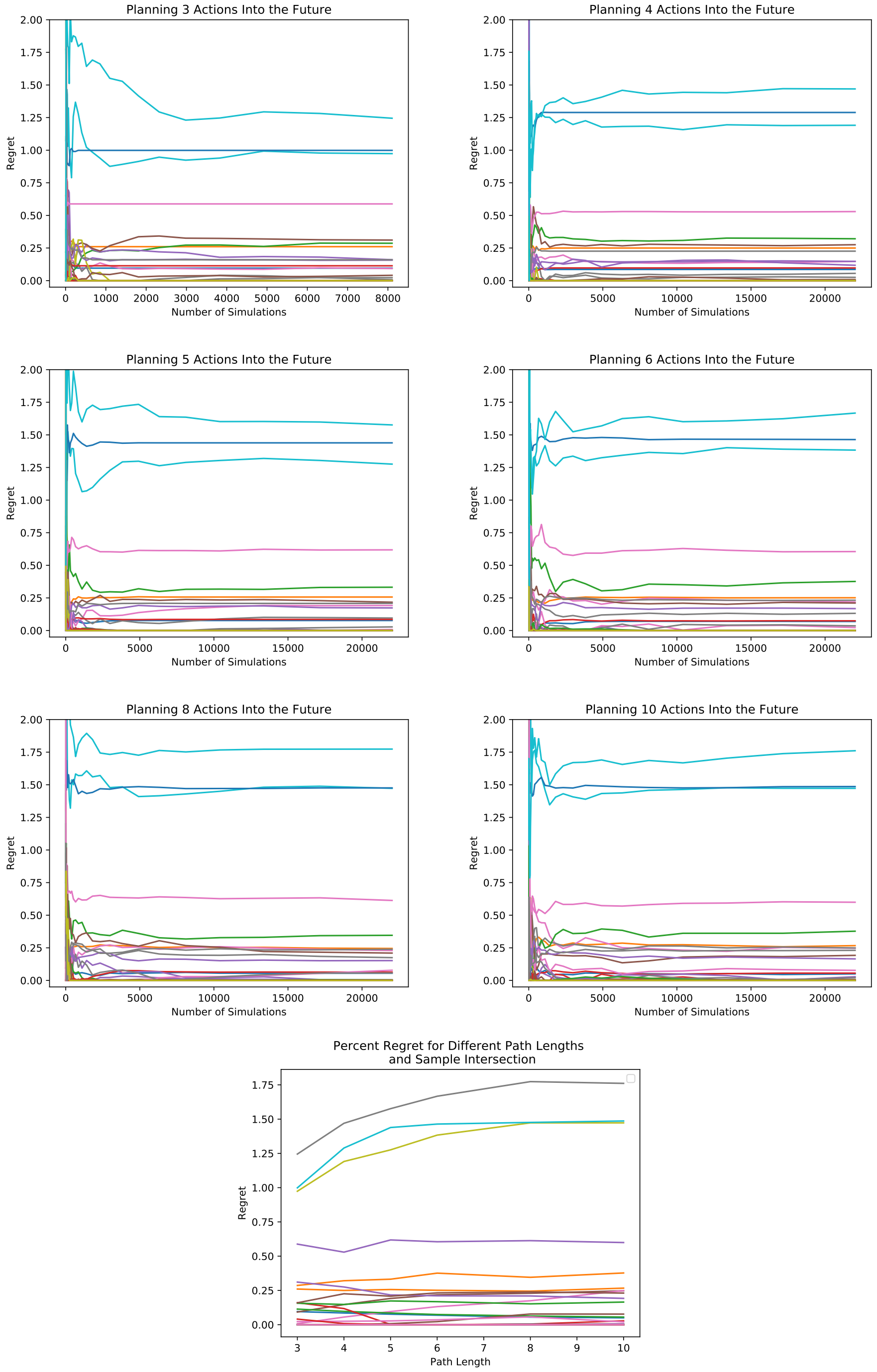


**Figure S14**: **Regret Convergence for the Optimal Agent in the Main Text.** The optimal agent is provided the same information as the participant. We plot the regret as we increase the number of simulations and actions (path length). One intersection is randomly selected for each experimental trial from three randomly selected participants (15, 24, and 41). Almost all trials have reached convergence for six actions and 10,000 simulations.

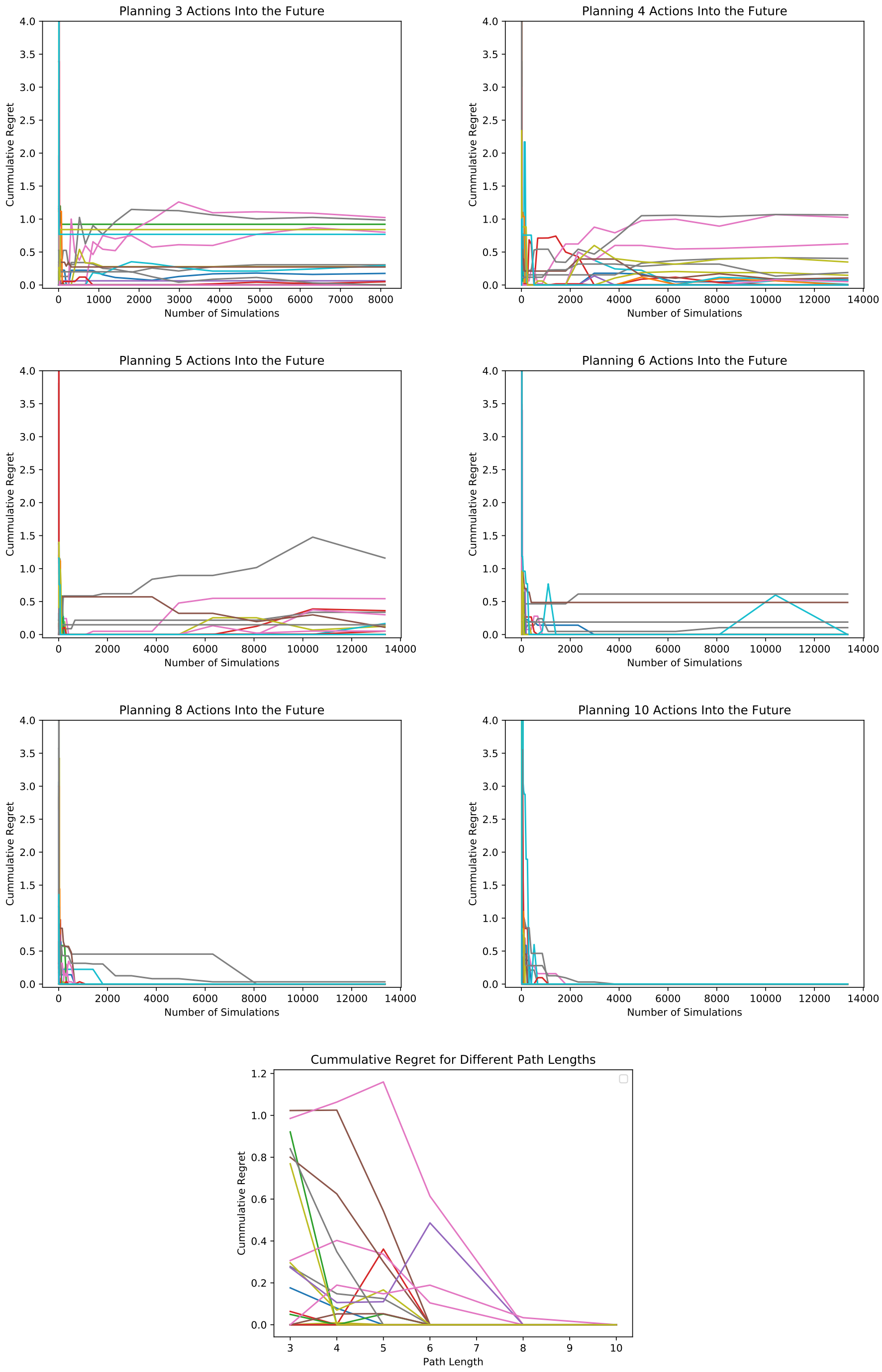


**Figure S15**: **Regret Convergence for the Optimal Agent with all Task Information**. The optimal agent with all task information is used to verify our simulated replica of the environment. We plot the regret as we increase the number of simulations and actions (path length). With a longer action horizon, the agent considers more paths and can often conceive of ways to recover from a poor short-term decision, resulting in smaller regret. One intersection is randomly selected for each experimental trial from three randomly selected participants (20, 8, and 36). Almost all trials have reached convergence for six actions and 10,000 simulations.

## 3 Supplementary References


[S1] Piantadosi, S.: Clinical Trials: a Methodologic Perspective. John Wiley & Sons, New York (2017)

[S2] Popovic, K., Schlafly, M.: Virtual Reality Code For "Collaborative Robots Can Augment Human Cognition in Regret-Sensitive Tasks" (2023). https://doi.org/10.5281/zenodo.10377640

[S3] Schlafly, M.: Haptics Code For "Collaborative Robots Can Augment Human Cognition in Regret-Sensitive Tasks" (2023). https://doi.org/10.5281/zenodo.10374409

[S4] Schlafly, M., Prabhakar, A.: Control Algorithm Code For "Collaborative Robots Can Augment Human Cognition in Regret-Sensitive Tasks" (2023). https://doi.org/10.5281/zenodo.10377309

[S5] Schlafly, M., Popovic, K., Schlafly, G.: Data Analysis Code For "Collaborative Robots Can Augment Human Cognition in Regret-Sensitive Tasks" (2023). https://doi.org/10.5281/zenodo.10377271

[S6] Schlafly, M.: MDP Code For "Collaborative Robots Can Augment Human Cognition in Regret-Sensitive Tasks" (2023). https://doi.org/10.5281/zenodo.10374434

[S7] Schlafly, M.: Dataset For "Collaborative Robots Can Augment Human Cognition in Regret-Sensitive Tasks" [Data set] (2023). https://doi.org/10.5281/zenodo.10373665

[S8] Tanvas - Surface haptic technology and products. https://tanvas.co/ (2021)

[S9] Wiertlewski, M., Fenton Friesen, R., Colgate, J.E.: Partial squeeze film levitation modulates fingertip friction. Proceedings of the National Academy of Sciences **113**(33), 9210–9215 (2016)

[S10] Mathew, G., Mezić, I.: Metrics for ergodicity and design of ergodic dynamics for multi-agent systems. Physica D: Nonlinear Phenomena **240**(4-5), 432–442 (2011)

[S11] Miller, L.M., Silverman, Y., MacIver, M.A., Murphey, T.D.: Ergodic exploration of distributed information. IEEE Transactions on Robotics **32**(1), 36–52 (2015)

[S12] Ayvali, E., Salman, H., Choset, H.: Ergodic coverage in constrained environments using stochastic trajectory optimization. International Conference on Intelligent Robots and Systems, 5204–5210 (2017). IEEE

[S13] Mavrommati, A., Tzorakoleftherakis, E., Abraham, I., Murphey, T.D.: Real-time area coverage and target localization using receding-horizon ergodic exploration. IEEE Transactions on Robotics **34**(1), 62–80 (2017)

[S14] Abraham, I., Murphey, T.D.: Decentralized ergodic control: distribution-driven sensing and exploration for multiagent systems. Robotics and Automation Letters **3**(4), 2987–2994 (2018)

[S15] Prabhakar, A., Abraham, I., Taylor, A., Schlafly, M., Popovic, K., Diniz, G., Teich, B., Simidchieva, B., Clark, S., Murphey, T.: Ergodic specifications for flexible swarm control: From user commands to persistent adaptation. Robotics: Science and Systems (2020)

[S16] Meyer, J., Prabhakar, A., Pinosky, A., Abraham, I., Taylor, A., Schlafly, M., Popovic, K., Diniz, G., Teich, B., Simidchieva, B., Clark, S., Murphey, T.: Scale-invariant specifications for human-swarm systems. Robotics: Science and Systems (2020)

[S17] Prabhakar, A., Murphey, T.: Mechanical intelligence for learning embodied sensor-object

relationships. Nature Communications **13**(1), 4108 (2022)

[S18] Losnegård, S.: Europe's largest drone operation after deadly landslide in norway. 420 missions and 200 hours of airtime. UAS Norway (2021)

[S19] Fernandes, O., Murphy, R., Adams, J., Merrick, D.: Quantitative data analysis: CRASAR small unmanned aerial systems at hurricane harvey. International Symposium on Safety, Security, and Rescue Robotics, 1–6 (2018)

[S20] Fernandes, O., Murphy, R., Merrick, D., Adams, J., Hart, L., Broder, J.: Quantitative data analysis: Small unmanned aerial systems at Hurricane Michael. International Symposium on Safety, Security, and Rescue Robotics, 116–117 (2019)

[S21] Mehta, R.K., Nuamah, J., Peres, S.C., Murphy, R.R.: Field methods to quantify emergency responder fatigue: lessons learned from sUAS deployment at the 2018 Kilauea volcano eruption. IISE Transactions on Occupational Ergonomics and Human Factors, 1–9 (2020)

[S22] Loianno, G., Mulgaonkar, Y., Brunner, C., Ahuja, D., Ramanandan, A., Chari, M., Diaz, S., Kumar, V.: Autonomous flight and cooperative control for reconstruction using aerial robots powered by smartphones. The International Journal of Robotics Research **37**(11), 1341–1358 (2018)

[S23] Szafir, D., Mutlu, B., Fong, T.: Designing planning and control interfaces to support user collaboration with flying robots. The International Journal of Robotics Research **36**(5-7), 514–542 (2017)

[S24] Chiou, M., Hawes, N., Stolkin, R.: Mixed-initiative variable autonomy for remotely operated mobile robots. ACM Transactions on Human-Robot Interaction (THRI) **10**(4), 1–34 (2021)

[S25] Zhou, J., Zhu, H., Kim, M., Cummings, M.L.: The impact of different levels of autonomy and training on operators' drone control strategies. ACM Transactions on Human-Robot Interaction **8**(4), 1–15 (2019)

[S26] Dorneich, M.C., Letsu-Dake, E., Singh, S., Scherer, S., Chamberlain, L., Bergerman, M.: Mixed-initiative control of a roadable air vehicle for non-pilots. Journal of Human-Robot Interaction **4**(3), 38–61 (2015)

[S27] Cacace, J., Finzi, A., Lippiello, V., Furci, M., Mimmo, N., Marconi, L.: A control architecture for multiple drones operated via multimodal interaction in search & rescue mission. International Symposium on Safety, Security, and Rescue Robotics, 233–239 (2016). IEEE

[S28] Chung, S.-J., Paranjape, A.A., Dames, P., Shen, S., Kumar, V.: A survey on aerial swarm robotics. IEEE Transactions on Robotics **34**(4), 837–855 (2018)

[S29] Qian, D., Xi, Y.: Leader–follower formation maneuvers for multi-robot systems via derivative and integral terminal sliding mode. Applied Sciences **8**(7), 1045 (2018)

[S30] Music̀, S., Hirche, S.: Control sharing in human-robot team interaction. Annual Reviews in Control **44**, 342–354 (2017)

[S31] Clark, S., Usbeck, K., Diller, D., Schantz, R.E.: CCAST: A framework and practical deployment of heterogeneous unmanned system swarms. GetMobile: Mobile Computing and Communications **24**(4), 17–26 (2021)

[S32] Franchi, A., Secchi, C., Ryll, M., Bulthoff, H.H., Giordano, P.R.: Shared control: Balancing autonomy and human assistance with a group of quadrotor UAVs. IEEE Robotics & Automation Magazine **19**(3), 57–68 (2012)

[S33] Sutton, R.S., Barto, A.G.: Reinforcement Learning: An Introduction. MIT press, Cambridge, MA (2018)

[S34] Kurniawati, H.: Partially observable markov decision processes and robotics. Annual Review of Control, Robotics, and Autonomous Systems **5**, 253–277 (2022)

[S35] Shalev-Shwartz, S., *et al.*: Online learning and online convex optimization. Foundations and Trends® in Machine Learning **4**(2), 107–194 (2012)

[S36] He, Q., Liu, J.L., Eschapasse, L., Beveridge, E.H., Brown, T.I.: A comparison of reinforcement learning models of human spatial navigation. Scientific Reports **12**(1), 1–11 (2022)

[S37] Mugan, U., MacIver, M.A.: Spatial planning with long visual range benefits escape from visual predators in complex naturalistic environments. Nature Communications **11**(1), 1–14 (2020)

[S38] Schrum, M.L., Johnson, M., Ghuy, M., Gombolay, M.C.: Four years in review: Statistical practices of likert scales in human-robot interaction studies. Companion of the 2020 ACM/IEEE International Conference on Human-Robot Interaction, 43–52 (2020)

[S39] Stapel, J., Mullakkal-Babu, F.A., Happee, R.: Automated driving reduces perceived workload, but monitoring causes higher cognitive load than manual driving. Transportation research part F: traffic psychology and behaviour **60**, 590–605 (2019)

[S40] Rushworth, M.F., Behrens, T.E.: Choice, uncertainty and value in prefrontal and cingulate cortex. Nature neuroscience **11**(4), 389–397 (2008)

[S41] De Waard, D.: The measurement of drivers' mental workload (1996)

[S42] Jafari, M.J., Khosrowabadi, R., Khodakarim, S., Mohammadian, F.: The effect of noise exposure on cognitive performance and brain activity patterns. Macedonian Journal of Medical Sciences **7**(17), 2924 (2019)

[S43] Knight, M.J., Tlauka, M.: Interactivity in map learning: The effect of cognitive load. Spatial Cognition & Computation **17**(3), 185–198 (2017)
